# Comparative Analysis between Synthetic Vitamin E and Natural Antioxidant Sources from Tomato, Carrot and Coriander in Diets for Market-Sized *Dicentrarchus labrax*

**DOI:** 10.3390/antiox11040636

**Published:** 2022-03-26

**Authors:** Ricardo Pereira, Mónica Costa, Cristina Velasco, Luís M. Cunha, Rui C. Lima, Luís F. Baião, Sónia Batista, Alexandra Marques, Tiago Sá, Débora A. Campos, Miguel Pereira, Diva Jesus, Sergio Fernández-Boo, Benjamin Costas, Manuela Pintado, Luisa M. P. Valente

**Affiliations:** 1 CIIMAR, Centro Interdisciplinar de Investigação Marinha e Ambiental, Universidade do Porto, Terminal de Cruzeiros do Porto de Leixões, Av. General Norton de Matos, S/N, 4450-208 Matosinhos, Portugal; ricardo.pereira@ciimar.up.pt (R.P.); mcmonicasiren28@gmail.com (M.C.); cvelasco@ciimar.up.pt (C.V.); luis.baiao@sensetest.pt (L.F.B.); batista_sonia@hotmail.com (S.B.); amarques@ciimar.up.pt (A.M.); tsa@ciimar.up.pt (T.S.); sboo@ciimar.up.pt (S.F.-B.); bcostas@ciimar.up.pt (B.C.); 2 ICBAS, Instituto de Ciências Biomédicas Abel Salazar, Universidade do Porto, Rua Jorge de Viterbo Ferreira, 228, 4050-313 Porto, Portugal; 3 CBQF–Centro de Biotecnologia e Química Fina—Laboratório Associado, Escola Superior de Biotecnologia, Universidade Católica Portuguesa, Rua Diogo Botelho 1327, 4169-005 Porto, Portugal; dcampos@ucp.pt (D.A.C.); miguelfpereira@gmail.com (M.P.); djesus@ucp.pt (D.J.); mpintado@ucp.pt (M.P.); 4 GreenUPorto/INOV4Agro & DGAOT, Faculdade de Ciências da Universidade do Porto, 4169-007 Porto, Portugal; lmcunha@fc.up.pt; 5 Sense Test Lda., R. Zeferino Costa 341, 4400-345 Vila Nova de Gaia, Portugal; rcl@sensetest.pt

**Keywords:** circular economy, European sea bass, functional aquafeeds, natural antioxidants, vitamin E, carotenoids, polyphenols, antioxidant activity

## Abstract

Synthetic vitamin E is commonly used in aquafeeds to prevent oxidative stress in fish and delay feed and flesh oxidation during storage, but consumers’ preferences tend towards natural antioxidant sources. The potential of vegetable antioxidants-rich coproducts, dried tomato (TO), carrot (CA) and coriander (CO) was compared to that of synthetic vitamin E included in diets at either a regular (CTRL; 100 mg kg^−1^) or reinforced dose (VITE; 500 mg kg^−1^). Natural antioxidants were added at 2% to the CTRL. Mixes were then extruded and dried, generating five experimental diets that were fed to European sea bass juveniles (114 g) over 12 weeks. Vitamin E and carotenoid content of extruded diets showed signs of degradation. The experimental diets had very limited effects on fish growth or body composition, immunomodulatory response, muscle and liver antioxidant potential, organoleptic properties or consumer acceptance. Altogether, experimental findings suggest that neither a heightened inclusion dose of 500 mg kg^−1^ of vitamin E, nor a 2% inclusion of natural antioxidants provided additional antioxidant protection, compared to fish fed diets including the regular dose of 100 mg kg^−1^ of vitamin E.

## 1. Introduction

Certain aquaculture practices, such as overcrowding, periodic handling, sudden changes in temperature and poor nutritional quality, generate stress in reared fish [1]. These stress situations could become ameliorable through the action of exogenous antioxidants, as their intake may mitigate oxidative damage by inhibiting the initiation or propagation of oxidative chain reactions [2,3]. Moreover, aquafeeds are usually rich in long chain polyunsaturated fatty acids (LC-PUFA), making them highly susceptible to lipid oxidation [4]. In fish fillet, rancidity and fatty acid depletion are also promoted by the presence of highly unsaturated fatty acids, leading to a deterioration of fillet quality over time, albeit at different levels depending on species, age and diet composition [5]. Thus, maintaining taste, colour, texture and freshness during storage is also a prime concern of the aquaculture industry [6].

Given the increasing importance of aquaculture production, which represents 53% of total fish supply for human consumption [7], the aforementioned issues associated with oxidative stress generate a rising necessity for the development of novel functional diets with physiological benefits beyond those provided by traditional feeds, particularly antioxidant benefits [8,9]. Due to their radical-scavenging properties and/or capacity of antioxidant system modulation, synthetic antioxidants are often used as additives for improving fish resistance to oxidative stress, as well as avoiding oxidative rancidity of fats and oils in feed mixtures [10,11]. Synthetic antioxidant compounds such as ethoxyquin, butylated hydroxyanisole (BHA) and butylated hydroxytoluene (BHT) are amongst the most widely used antioxidants in food and feed formulations [4]. In 2017, the common use of ethoxyquin was banned in raw materials, feed premixes, additives and food produced and/or commercialized in the European Union (EU 2017/962). This occurred due to possible genotoxicity effects of one of its metabolites (ethoxyquin quinone imine), as well as the fact that p-phenetidine, an impurity of ethoxyquin, is recognised as a possible mutagen [12]. Meanwhile, BHT (E321) and BHA (E320) are both widely used as antioxidants in feed for all animal species, with a maximum limit of 150 mg kg^−1^ each, or in combination, authorised in the EU [13]. Nevertheless, BHT has been found to inhibit humoral immune response in animals and to possess tumour promotion effects, and despite not being genotoxic, it modifies the genotoxicity of other agents [14]. This, together with the proven effectiveness of natural antioxidants for inhibiting rancidity in food [15] and in providing health advantages for consumers [16,17], prompted the scientific community and the feed industry to look for natural alternatives with strong anti-oxidant activity for inclusion in animal feeds [9,18,19,20,21].

Vitamins, carotenoids and phenolic compounds are antioxidant molecules commonly present in fruits, herbs and vegetables such as tomato, carrot and coriander [22,23,24]. Specifically, vitamin E is a generic term commonly used for describing eight different lipid-soluble antioxidant forms (tocopherols and tocotrienols), the form of α-tocopherol having the greatest impact on the fish antioxidant system [25]. These compounds are easily oxidized due to their inherent hydroxyl moiety on carbon 6, which has an important role in the maintenance of normal metabolic processes and physiological functions in the body, mainly due to its radical-scavenging properties [26,27,28], thus playing an important role in the protection of cell membranes from lipid peroxidation [29,30]. Vitamin E also shows a stimulatory effect on fish’s immune system, while improving fish health and disease resistance [31,32]. This antioxidant compound has been proven to have an essential protective role against the adverse effects of reactive oxygen species and other free radicals [4], which is important for quality preservation of fish fillet, either raw or cooked. To date, the positive influence of vitamin E on seafood quality has been studied in several commercially reared fish species, such as rainbow trout (*Oncorhynchus mykiss*) [33], turbot (*Scophthalmus maximus*) [34] and European sea bass (*Dicentrarchus labrax*) [35]. The minimum recommended doses of vitamin E in fish dietary mixes range from 25 to 200 mg kg^−1^, depending on the species and maturation state [4]. Although vitamin E supplementation has been demonstrated as essential for the development of European sea bass larvae [36], very few data are available regarding the antiperoxidative effects of vitamin E in commercial-sized European sea bass. Gatta et al. [35] suggested that the α-tocopherol content of diets for European sea bass (208 g) should be above 254 mg kg^−1^, and up to 942 mg kg^−1^, in order to reduce lipid oxidation.

Phenolic compounds and carotenoids are also associated with a wide range of biological activities, including antioxidant properties that contribute both directly and indirectly to the inhibition or suppression of oxidation processes [17,37]. Carotenoid-rich diets are effective in terms of preventing oxidative stress and enhancing innate immune system defences. Ehsani et al. [21] found that dietary lycopene, naturally abundant in carrots and tomatoes, is effective in preventing lipid oxidation in rainbow trout, subsequently delaying fillet rancidity. β-carotene, found in high doses in carrot and coriander acted as an antioxidant and immunostimulant, mitigating the negative effects of peroxide radicals in Nile tilapia (*Oreochromis niloticus*) [18]. Both synthetic and natural carotenoid dietary inclusion has been proven to stimulate antioxidant potential in European sea bass [6,38,39], while immunomodulatory effects were reported with the dietary inclusion of carotenoid rich sources such as seaweed [38].

Annual production of plants and aromatic herbs has passed 600 million tonnes per year [39], generating a high amount of coproducts that are mostly wasted. Despite viability for consumption, about a third of total farm production in the EU is discarded, mostly due to cosmetic and retailers’ standards [40]. These otherwise discarded coproducts can be valuable sources of nutrients, antioxidants and bioactive compounds for aquafeeds that at a reduced price may have a positive effect on weight gain, appetite stimulation and overall fish health [41,42,43,44], as well as in fillet quality traits [17,21]. Adding these vegetable coproducts to aquafeeds would also address current consumer trends in terms of preferring natural food sources over synthetic ones [45], and promote a circular economy approach towards sustainability.

The novel approach of this study consisted of evaluating the potential of vegetable antioxidants-rich coproducts as additional antioxidant sources in diets for European sea bass, a carnivorous fish of high economical value, commonly reared in the Mediterranean region. The effects of such natural antioxidants on European sea bass growth, immune system and fillet quality traits will be compared to those of synthetic vitamin E included in diets at either a regular or reinforced dose (100 and 500 mg kg^−1^, respectively).

## 2. Materials and Methods

### 2.1. Ingredients and Experimental Diets

Fresh carrot (*Daucus carota* subsp. *Sativus*), coriander (*Coriandrum sativum*) and tomato (*Solanum lycopersicum*) biomass were obtained from VITACRESS PORTUGAL, S.A., one of Europe’s leading companies in the delivery of fresh, washed and ready-to-eat vegetables. Vegetables were lyophilized, packed in vacuum and stored under refrigeration at −20 °C, until use. Proximate composition, main carotenoids, vitamin E and antioxidant potential are described in Table 1. A commercial-based diet for European sea bass was formulated relying on practical ingredients (Table 2) and supplemented with a commercial vitamin premix that contained a regular dose of vitamin E (α-tocopherol, 100 mg kg^−1^) as part of the vitamin and mineral premix. This diet was used as a negative control (CTRL) and compared with a positive control diet (VITE) containing an additional amount of added α-tocopherol (Lutavit^®^ E50), totalling 500 mg kg^−1^. The three experimental diets were obtained after adding 2% of each dried biomass from the three natural antioxidant sources to the CTRL mix, at the expense of wheat meal: carrot (CA), tomato (TO) and coriander (CO). Diets were grounded, extruded (110 °C, originating 4 mm pellets) and dried (60 °C until <8% of moisture) by SPAROS, LDA. (Portugal). All diets were kept isoproteic (49% of dry matter, DM), isolipidic (18% DM) and isoenergetic (23 kJ g^−1^ DM). At the beginning of the trial, 10 g of each experimental diet and ingredient were frozen at −80 °C for proximate composition analysis and antioxidant profile, including carotenoid quantification and profile, vitamin E and hydrolysable polyphenol quantification, as well as antioxidant potential through the radical scavenging potential of 2,2′-azino-bis-3-ethylbenzthiazoline-6-sulphonic acid (ABTS^•+^) and 1,1-diphenyl-2-picrylhydrazyl (DPPH^•^).

### 2.2. Growth Trial and Fish Rearing Conditions

The fish trial and all animal procedures were subject to an ethical review process carried out by CIIMAR animal welfare body (ORBEA-CIIMAR_18_2017) and further approved by the Portuguese Veterinary Authority (1005/92, DGAV-Portugal), in compliance with the guidelines of the European Union (directive 2010/63/UE). European sea bass juveniles obtained from a commercial fish farm (ACUINUGA, A Coruña, Spain) were transported to the Fish Culture Experimental Unit of CIIMAR (Matosinhos, Portugal). Fish were kept in quarantine for two weeks. After acclimation, fish were fasted for 24 h, anesthetized with 2-phenoxyetanol (200 μL L^−1^), and individually weighed (g) and measured (total length, cm). Homogeneous groups of 20 fish (initial body weight of 114 ± 0.2 g; initial length of 22 ± 0.1 cm) were randomly distributed by 250 L fibreglass tanks within a saltwater recirculation system with water flow rate adjusted to 16 L h^−1^. Water quality parameters were continuously monitored, and temperature was maintained at 22 ± 1.0 °C, salinity at 35 ± 0.5‰ and water oxygen levels at a minimum 90% saturation. Redox potential, pH levels and salinity were measured daily. Total ammonium, nitrate and nitrite were monitored twice a week and maintained at levels ≤0.05 mg L^−1^, ≤0.5 mg L^−1^ and ≤50 mg L^−1^, respectively, as is recommended for marine species [46]. A 12 h photoperiod was settled. Each experimental diet was tested in triplicate tanks, and fish were manually fed until apparent visual satiation, twice a day for 87 days.

Ten fish from the initial stock and five fish per tank by the end of the feeding trial were sacrificed by an anaesthetic overdose (2-phenoxyethanol, 500 μL L^−1^) and stored at −80 °C for assessing whole-body composition. An intermediate weighting was performed mid-trial in order to assess fish growth and feed conversion ratio (FCR). At the end of the growth trial, after a 24 h fasting period, fish were slightly anesthetized with 2-phenoxyetanol (200 μL L^−1^) and were individually weighed (g) and measured (total length, cm) for determination of growth rate. Blood was collected from four fish per tank, taken from the caudal vein using heparinized syringes, and centrifuged (10,000× *g* for 5 min at 4 °C); the resulting plasma was stored at −80 °C for analysing innate immune parameters. These four fish were then sacrificed by a sharp blow on the head, after which intestine and liver were weighed for determination of the viscerosomatic and hepatosomatic indexes. Afterwards, liver and left dorsal muscle samples from each fish were taken, immediately frozen in liquid nitrogen, and kept at −80 °C until analyses. Oxidative stress enzyme activity, lipid peroxidation and total antioxidant capacity, as well as chemical composition parameters, namely moisture, total lipids and ash, were performed in the liver; while left dorsal muscle was sampled for vitamin E quantification, antioxidant potential (DPPH^•^ and ABTS^•+^), lipid peroxidation and chemical composition analyses. Additionally, the right dorsal fillet was collected for immediate instrumental colour analyses of skin and muscle. Instrumental determination of right dorsal muscle texture was also performed at this time point (Day 0).

Four additional fish per tank were sacrificed by an anaesthetic overdose using 2-phenoxyethanol, and subsequently stored in Styrofoam boxes with ice and kept at 4 °C, protected from light. After 8 days on ice, the right dorsal fillet was collected for analysing instrumental colour and texture determination. Moreover, similarly to Day 0, right dorsal muscle was sampled for measuring the antioxidant potential (DPPH^•^ and ABTS^•+^) and lipid peroxidation.

Finally, another seven fish per tank were fasted for 48 h, sacrificed by ice slurry and placed in Styrofoam boxes for 24 h before sensory analyses.

### 2.3. Chemical Composition

Whole fish and fish tissues were ground, homogenised and freeze dried before analyses. Proximate composition was performed in accordance with AOAC methods [47]. All samples were analysed in duplicates for dry matter (DM) (105 °C for 24 h); ash, through muffle furnace combustion at 500 °C (5 h) (Nabertherm L9/11/B170, Bremen, Germany); crude protein (N × 6.25), using a Leco nitrogen analyser (Model FP 528; Leco Corporation, St. Joseph, MO, USA); and crude fat (petroleum ether extraction), using a Soxtec extractor (Model ST 2055 SoxtecTM; FOSS, Hillerod, Denmark), for whole fish. Total lipids were measured in muscle and liver, using a dichloromethane: methanol (2:1 wv^−1^) extraction followed by gravimetrical quantification [48]. Total phosphorus content was determined from ashes by digestion at 150 °C in hydrochloric acid, followed by the quantification of phosphates using ammonium molybdate at 75 °C in a water bath and later determination of absorbance at 820 nm according to ISO 13730:1996 [49]. Gross energy was determined in an adiabatic bomb calorimeter (Model Werke C2000, IKA, Staufen, Germany).

Carotenoids and α-tocopherol in ingredients and diets were analysed in duplicate, using extracts obtained in accordance with Slavin and Yu [50], with slight modifications. Briefly, 100 mg of each sample were mixed with 3 mL of ethanol, ground in Ultra-Turrax for 2 min, and mixed with 8 mL of n-hexane, re-ground and centrifuged, after which the supernatant was collected. After this, the supernatant was mixed with 0.1% ascorbic acid (wv^−1^), vortexed and placed in an 85 °C water bath for 5 min. Afterwards, the mixture was cooled on ice, 5 mL of NaCl 1 M and 8 mL of n-hexane were added, after which the solution was centrifuged at 1000× *g* for 5 min at 4 °C, and the supernatant collected. After repeating the last step, the final hexane extraction volume was washed with 5 mL of 5% Na_2_CO_3_ (wv^−1^) and centrifuged at 1000× *g* for 5 min at 4 °C. The resulting supernatant was washed with 5 mL of ultrapure water and evaporated under nitrogen gas steam, after which it was dissolved in isopropanol and frozen at −20 °C. This extract was used for carotenoid quantification and identification, performed according to Gómez-García et al. [51], through HPLC (Waters Series 600, Mildford, MA, USA), using acetonitrile, methanol, dichloromethane, hexane and ammonium acetate (55:22:11.5:11.5:0.02 *v/v/v/v/w*) under isocratic conditions at 1 mL min^−1^ flow rate during 20 min at 30 °C. Injection volume was 50 μL and detection was performed by a 454 nm diode array detector (Waters, Massachusetts, EUA). α-carotene, β-carotene, lutein, lycopene and β-cryptoxanthin were quantified using a pure standard calibration curve expressed as mg 100 g^−1^ DM. Analysis of α-tocopherol in ingredients and diets was performed via chromatography, using a Beckman System Gold^®^ HPLC system (Beckman Coulter, Pasadena, CA, USA) linked to a Waters™ 474 Scanning Fluorescence Detector (excitation wavelength of 290 nm and emission wavelength of 320 nm) with a VARIAN ProStar Model 410 AutoSampler with a normal-phase silica column (Kromasil 60-5-SIL, 250 mm, 4.6 mm ID, 5 µm particle size). The mobile phase was 1% (vv^−^^1^) isopropanol in n-hexane with a flow rate of 1 mL min^−1^. The total run time was 20 min and the injection volume was 20 µL. Standard curves of peak area vs. concentration were used for each compound quantification.

Total phenolic compounds were determined in ingredients and diets, in duplicate, according to the method described by Xie et al. [52], with some modifications. Briefly, 1 g of each ingredient and diet was washed with distilled water under stirring for 30 min at room temperature to remove soluble and free phenolic compounds. This extract was centrifuged at 1000× *g* for 10 min, after which the supernatant was dissolved in pure methanol (5 mL). Measurement was performed according to the Folin–Ciocalteu method at 750 nm (Synergy H1 HU126, Winooski, VT, USA).

### 2.4. Innate Immune Parameters and Plasma Bactericidal Activity

In fish plasma, the activities of lysozyme, peroxidase and alternative complement pathway (ACH50) were measured using a microplate spectrophotometer (BioTek Synergy HT, Winooski, VT, USA), in triplicate. Lysozyme activity was measured using a turbidimetric assay as described by [53]. A *Micrococcus lysodeikticus* solution (0.5 mg mL^−1^, 0.05 M sodium phosphate buffer, pH 6.2) was prepared. Then, 15 µL of plasma was added, in triplicates, to a microplate and 250 µL of the above suspension were pipetted to give a final volume of 265 µL. The reaction occurred at 25 °C, after which absorbance (450 nm) was measured after 0.5 and 4.5 min. A standard curve was generated using a serial dilution of lyophilized hen egg white lysozyme (Sigma) in sodium phosphate buffer (0.05 M, pH 6.2. The amount of lysozyme in the sample was calculated using the standard curve. Total peroxidase activity (EU mL^−1^ plasma) was measured according to Quade and Roth [54], adapted by Costas et al. [53], and was determined by defining that one unit of peroxidase produces an absorbance change of 1 OD. Alternative complement pathway (ACH50) activity was determined based on the lysis of rabbit red blood cells (2.8 × 108 cells mL^−1^; Probiológica, Belas, Portugal) in the presence of ethylene glycol tetra acetic acid (EGTA; Sigma) and Mg^2+^ (MgCl_2_·^6^H_2_O; VWR), as described by Sunyer and Tort [55]. ACH50 units were defined as the concentration of plasma giving 50% lysis of cells.

Plasma levels of immunoglobulin M (IgM) were analysed using an enzyme-linked immunosorbent assay (ELISA), in accordance with Costa et al. [56]. Essentially, flat-bottomed 96-well plates were coated overnight with European sea bass plasma, diluted at 1:100 using 50 mM carbonate bicarbonate buffer (pH 9.6). Samples were then blocked with 300 μL powdered low fat milk, diluted at 5% wv^−1^ in T-TBS (20 mM Tris Base, 137 mM NaCl and Tween 20 at 1% vv^−1^) for 1 h, after which they were cleaned 3 times with T-TBS solution and subsequently incubated for 1 h with 100 μL of primary antibody (mouse anti-European sea bass IgM monoclonal antibody, 1:100 in blocking buffer; Aquatic Diagnostics Ltd., Scotland, UK). Samples were cleaned 3 times with T-TBS solution after incubation, and incubated again with 100 μL of the secondary antibody anti-mouse IgG-HRP (1:1000 in blocking buffer, Sigma-Aldrich, Darmstadt). After this incubation, samples were cleaned with T-TBS and 100 μL of 3,30,5,50-tetramethylbenzidine hydrochloride (TMB, Sigma—Aldrich, Darmstadt, Germany) at 1 mg mL^−1^ was added. After 5 min incubation, the reaction was stopped with 100 μL of H_2_SO_4_ 2M, and absorbance was measured at 450 nm on a Synergy HT microplate reader (BioTek Synergy HT, Winooski, VT, USA). Negative controls consisted of HBSS instead of plasma. OD values were subtracted for each sample value.

Bactericidal activity in fish plasma was measured according to Costa et al. [56]. *Vibrio anguillarum* (VA) and *Photobacterium damselae* subsp. *piscicida* (Pdp) were cultured in tryptic soy broth (TSB; Difco). For assessing bactericidal activity, 20 μL of plasma was diluted in 20 μL of the previously mentioned bacterial suspensions, in duplicate, at a concentration of 1 × 10^6^ cfu/mL, into a U-shaped 96-well microplate. The resulting mixture was incubated for 3 h at 25 °C with shaking (100 rpm). Afterwards, 25 μL of 3-(4, 5 dimethyl-2-yl)-2,5-diphenyl tetrazolium bromide (MTT, 1 mg mL^−1^; Sigma) was added. The solution was then incubated for 10 min at 25 °C with shaking (100 rpm). Plates were centrifuged at 2000× *g* for 10 min, and the resulting pellet was dissolved with 200 μL of Dimethyl Sulfoxide (DMSO, Sigma—Aldrich, Darmstadt, Germany). The solutions (100 μL) were then placed into a flat-bottomed 96-well microplate and the absorbance was measured at 490 nm using a Synergy HT microplate reader (Biotek, VT, USA). Bactericidal activity was calculated as the difference between bacterial surviving and positive control (100%). Results are expressed as the percentage of non-viable bacteria.

### 2.5. Antioxidant Potential and Oxidative Stress

Antioxidant capacity of ingredients, diets, as well as fish muscle, before and after 8 days on ice, was measured through assessment of the radical scavenging potential of ABTS^•+^ and DPPH^•^. For ABTS^•+^, radical scavenging activity was measured in the methanolic extracts using the method described by Sánchez-Moreno [57] and adapted by Gonçalves et al. [58]. Essentially, using a flat-bottom 96-well microplate, 180 µL of ABTS^•+^ working solution was added to 20 µL of sample (in triplicate). The mixture was incubated for 5 min at 30 °C, protected from light, and the absorbance at 734 nm was measured with a multi-detection plate reader (Synergy H1 HU126, Winooski, VT, USA). The DPPH^•^ assay was performed according to the method of Brand-Williams et al. [59], with some modifications. The assay was performed in a flat-bottomed 96-well microplate, to 25 µL of the sample, 175 µL of DPPH^•^ working solution was added, in triplicate. In both analytical procedures, Trolox was used for the standard curve and methanol 80% was used for the blanks, as it was the solvent used for the analysed extracts. The mixture was incubated for 30 min at 25 °C, protected from light, and the absorbance was measured at 515 nm with a multi-detection plate reader (Synergy H1 HU126, Winooski, VT, USA). For both analyses, final results were expressed in Trolox equivalents (TE) per 100 g of DM, or µg of TE per g wet weight (ww^−1^) in the case of fish muscle.

Liver samples of fish fed with the experimental diets were homogenized using phosphate buffer (0.1 M, pH 7.4), in a proportion of 1:10 (*w*:*v*). After this, samples were centrifuged at 10,000× *g* for 15 min at 4 °C, and the supernatant aliquoted and stored at −80 °C for determining the activity of oxidative stress enzymes. Protein content was determined in accordance with Bradford [60] for standardizing antioxidant enzyme activity measurements. The following analysis were carried out in triplicates using a microplate reader. Catalase (CAT) activity was measured according to Greenwald [61], using hydrogen peroxide (H_2_O_2_) 30% as substrate. Alterations in absorbance were registered at 240 nm, at 25 °C. CAT activity was calculated as µmol of H_2_O_2_ consumed per min per mg of protein. Glutathione s-transferase (GST) was determined according to Habig [62]. Essentially, total activity (cytosolic and microsomal) was determined by measuring the conjugation of 1-chloro-2,4-dinitrobenzene (CDNB) with reduced glutathione (GSH). Changes in absorbance were recorded at 340 nm, at 25 °C for 5 min, and enzyme activity was calculated as nmol of CDNB conjugate formed per min per mg of protein. Glutathione reductase (GR) activity was measured according to Cribb et al. [63], assessing NADPH disappearance at 340 nm for 3 min at 25 °C, and expressing the results in nmol of oxidized NADPH per minute, per mg of protein. Total glutathione (TG) was evaluated by measuring the formation of 5-thio-2-nitrobenzoic acid (TNB) at 412 nm, as detailed in Baker et al. [64], with results expressed as nmol of conjugated TNB formed per min per mg of protein. Glutathione peroxidase (GPx) was measured as reported by Mohandas et al. [65], through an indirect method based on the oxidation of reduced glutathione (GSH) to oxidized glutathione (GSSG) catalyzed by GPx. Reaction was performed at 25 °C with a pH of 8.0, using H_2_O_2_ as substrate and including sodium azide (NaN_3_) as a catalase inhibitor. Oxidation of NADPH was recorded spectrophotometrically at 340 nm at 25 °C, after which the enzyme activity was calculated as nmol of oxidized NADPH per min per mg of protein. Lipid peroxidation (LPO) was determined in concordance with Bird and Draper [66], by quantifying the presence of thiobarbituric acid reactive substances (TBARS), composed mainly of malondialdehyde (MDA). The decomposition of unstable peroxides derived from polyunsaturated fatty acids (PUFAs) induces the formation of MDA, subsequently quantified colorimetrically following its controlled reaction with thiobarbituric acid (TBA). The absorbance was measured at 535 nm, at 25 °C, and the rate of LPO was expressed as nmol of MDA formed per g of fresh tissue. The concentration of total antioxidant in samples was determined by using the total antioxidant capacity assay kit (Sigma MAK187), by measuring the formation of TE. Results were expressed in nmol per mg of tissue.

Muscle samples were homogenized using phosphate buffer (0.1 M, pH 7.4) in a proportion of 1:10 (*w*:*v*) and protein content determined according to Bradford [60]. Lipid peroxidation (LPO) was measured in muscle samples collected on both sampling days (Day 0 and Day 8), using the methodologies described for liver. LPO was expressed as nmol of TBARS formed per gram of fresh tissue, and a comparison of results was performed via two-way ANOVA between fish sampled at the end of the feeding trial and fish stored in ice for 8 days.

### 2.6. Instrumental Texture and Colour

Skin and muscle colour measurements were performed in two different groups of fish at Day 0 and Day 8, immediately after sampling in fish from Day 0, and after storage on ice for 8 days in fish from Day 8. Measurements were performed with a CR-400 chroma meter (Konica Minolta Inc., Osaka, Japan) with an aperture of 8 mm, with respect to CIE standard illuminant D65. The apparatus was calibrated using a white plate reference standard (Minolta Co, Ltd., Osaka, Japan). Colour parameters were measured by applying the colorimeter onto raw fillets from 12 fish per dietary treatment. Measurements were made in three points above the lateral line of each fillet. After flashing, L*, a* and b* reflected light values were recorded. From a* and b* values the hue angle (h* = tan^−1^ b*/a*) and the chroma (C* = (a*^2^ + b*^2^)^1/2^) were calculated according to Choubert et al. [67]. The same procedure was applied to fish that were stored in ice for 8 days (Day 8).

Muscle texture was also analysed in fish both before and after a storage period in ice for 8 days using a TA.XT.plus Texture Analyser (Stable Micro Systems Inc., Godalming, United Kingdom) with a 5 kg load cell and a 2.0 mm diameter probe. Texture profile parameters [hardness (N), adhesiveness (J), springiness (-), cohesiveness (-), chewiness (J) and resilience (-)] were obtained by double compression (constant speed and penetration depth of 1 mm s^−1^ 320 and 4.0 mm, respectively) on the thickest part of each raw fillet according to Batista et al. [68]. Penetration depth was selected according to the maximum distance that did not induce fibre breakage.

### 2.7. Consumer Acceptance

**Figure 1:**
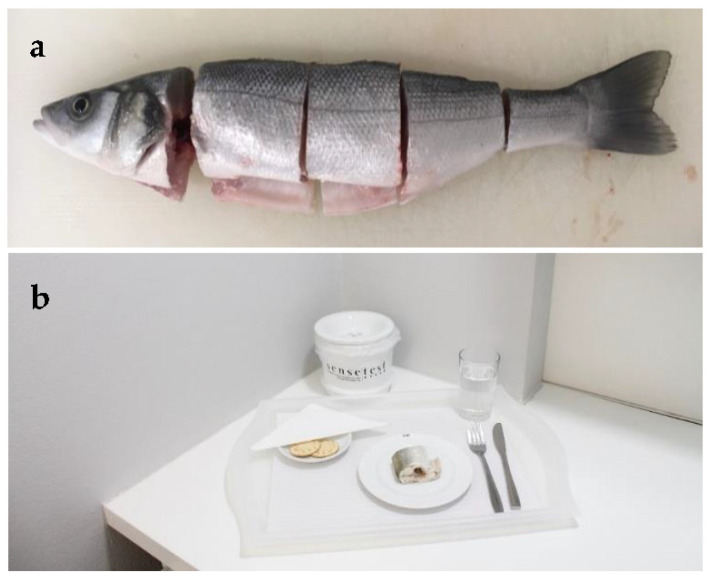
(**a**) Fresh sea bass descaled, gutted and sliced; (**b**) Individual slices served to participants after steam cooking wrapped in micro-perforated aluminium foil, presented in preheated white porcelain plates with a random three-digit code.

For sensory evaluation, European sea bass were sampled and prepared for evaluation after removal of gut and scales. Heads, tails and fins were cut-off and fish were cut into three slices (anterior, middle and posterior) (Figure 1a). Each slice was wrapped in micro-perforated aluminium foil and steamed for 12 min at 100 °C, in a preheated industrial forced convector oven with steam (Rational Combi-Master CM61, Rational AG, Germany). Each consumer received slices from the same part of the fish (anterior, middle or posterior) across all samples (Figure 1b).

The samples were served over white pre-heated (50 °C) porcelain plates, coded with a three-digit random number. Panellists were provided with cutlery, a glass of bottled natural water and unsalted crackers (Figure 1b). All panellists were asked to chew a piece of cracker and to rinse the mouth with water before testing each sample. To compensate for eventual carry-over effects, each panellist received the set of five samples following a monadic sequential presentation, with their order previously balanced, in accordance with MacFie et al. [69]. For each sample, a total of 60 naïve consumers evaluated overall liking using a 9-point hedonic scale, ranging from 1—“dislike extremely” to 9—“like extremely” [70]. For each sample, after the overall liking evaluation, each consumer was requested to make a comment regarding the sample, considering the main negative and positive aspects. The panel was recruited from the sensory evaluation company Sense Test’s consumer database (Vila Nova de Gaia, Portugal). They were mainly residents in the Oporto metropolitan area, in the North of Portugal. All participants were regular consumers of fish, at least two times per week. Besides the implementation of informed consent, the company ensures the protection and confidentiality of data through the authorization 2063/2009 of the National Data Protection Commission, and following EU Regulation (EU 2016/679), as well as a longstanding internal code of conduct. Sensory evaluation was carried in individual tasting booths in a special room equipped in accordance with ISO 8589:2007—sensory analysis—general guidance for the design of test rooms.

### 2.8. Calculations

Growth, Intake and Retention:

ABW = (FBW + IBW)/2

K = (FBW/final body length^3^) × 100

DGI = 100 × ((FBW)^1/3^ − (IBW)^1/3^)/days

VFI = 100 × crude feed intake/ABW/day

FCR = dry feed intake/weight gain

(DGI) = 100 × [(FBW) 1/3 − (IBW)1/3]/days

PER = weight gain/crude protein intake

P, L or E gain = (final carcass P, L or E content − initial carcass P, L or E content)/ABW/days;

Somatic Indexes:
HSI = 100 × liver weight/FBWVSI = 100 × viscera weight/FBWABW—Average body weightK—Fulton’s condition factor FBW—Final body weightDGI—Daily growth indexIBW—Initial body weightVFI—Voluntary feed intakeN—NitrogenFCR—Feed conversion ratioP—PhosphorousPER—Protein efficiency ratioL—LipidsHSI—Hepatosomatic index E—EnergyVSI—Viscerosomatic index

All calculations were performed according to NRC [4].

### 2.9. Statistical Analysis

All statistical analyses were performed with IBM SPSS STATISTICS, 25.0 package (IBM corporation, New York, NY, USA, 2017), with the exception of the correspondence analysis that was performed with XLSTAT v. 2020 [71].

Data were tested for normality and homogeneity of variances by Shapiro-Wilk and Levene’s tests, respectively, and transformed whenever required before being submitted to a one-way ANOVA. When this test showed significance, individual means were compared using HSD Tukey Test. When data did not meet the assumptions of ANOVA, a non-parametric test, Kruskal-Wallis test, was performed. When needed, the Mann–Whitney test was carried out to identify significant differences between groups. In all cases, the level of significance was set at 0.05. A two-way ANOVA, with dietary treatment and storage time (0 or 8 days) was used to compare fish muscle antioxidant potential, colour and texture, as well as skin colour.

For overall liking, a three-way mixed effects ANOVA, with panellists as random factor, and fish part (anterior, middle and posterior) and dietary treatment as fixed factors, with no interaction effect, was used to investigate differences between treatments [72]. For the open comments, a content analysis was performed counting the number of times that each attribute (positive and negative) was mentioned per sample. The frequency of mention of each attribute was determined, calculating the number of consumers who have used each attribute to describe the samples. Over this frequency matrix, a correspondence analysis (CA) was applied. Such analysis provides a sensory map of the samples, allowing the perception of the similarities and differences between samples and their sensory characteristics [73,74,75].

## 3. Results

### 3.1. Characterisation of Ingredients and Experimental Diets

Dried tomato, carrot and coriander were shown to be variable sources of α-carotene, β-carotene and lutein (Table 1). α-carotene was below the quantification limit in tomato, while β-carotene was below the quantification limit in tomato and carrot. Coriander displayed the highest levels of α-carotene, β-carotene and lutein (14.6, 57.3 and 124.5 mg 100 g^−1^ DM, respectively). Carrot showed the second highest amount of α-carotene (4.7 mg 100 g^−1^ DM), while tomato displayed the second highest amount of lutein (1.1 mg 100 g^−1^ DM). Moreover, certain carotenoids were exclusive to specific vegetables: tomato was the only tested source of lycopene (18.3 mg 100 g^−1^ DM), while coriander was the only ingredient containing β–cryptoxanthin (0.6 mg 100 g^−1^ DM). Concerning vitamin E, coriander was the highest source (48.3 mg α-tocopherol kg^−1^ DM), followed by tomato (32.6 mg kg^−1^ DM) and carrot (10.5 mg kg^−1^ DM).

In terms of total phenolic compounds content, carrot had the highest amount of polyphenols (2478.7 mg GAE 100 g^−1^ DM), followed by tomato (729.6 mg GAE 100 g^−1^ DM) and coriander (528 mg GAE 100 g^−1^ DM) (Table 1). However, ingredients’ ABTS^•+^ and DPPH^•^ values showed coriander to have significantly higher values (62.0 and 74.2 TE 100 g^−1^ DM, respectively), followed by carrot (104.9 and 47.2 TE 100 g^−1^ DM, respectively) and tomato (62.0 and 21.0 TE 100 g^−1^ DM, respectively).

As shown in Table 2, TO and CO diets showed the highest amounts of lutein (1.8 and 1.7 mg 100 g^−1^ of feed, respectively) followed by the CTRL (1.5 mg 100 g^−1^ of feed), CA (1.0 mg 100 g^−1^ of feed) and VITE (0.6 mg 100 g^−1^ of feed) diets. The levels of α-tocopherol quantified in diets, after extrusion, corresponded well to the amounts of synthetic vitamin E included in each experimental diet: VITE diet had five times more α-tocopherol than any other diet. However, quantified values after extrusion were four times lower than those initially added to each diet. The CA diet contained the highest amount of total phenolic compounds (1179.1 mg GAE 100 g^−1^ of DM), followed by CO and TO (536.0 mg and 537.9 GAE 100 g^−1^ of DM, respectively). Out of all diets, the CO diet was shown to have the highest DPPH^•^ scavenging capacity (16.6 1.1 mg TE 100 g^−1^ of feed), in concordance with the higher DPPH^•^ values found in coriander when compared to the remaining ingredients.

### 3.2. Effects on Growth Performance, Whole Body Composition, Innate Immune Parameters and Plasma Bactericidal Activity

All diets were well accepted by fish, with no significant differences in final body weight, leading to a similar growth performance (DGI) and feed efficiency between groups (Table 3). Voluntary feed intake (VFI) was similar between all treatments, as well as final body composition and nutrient gain (Table 3). Hepatosomatic and viscerosomatic indexes and liver chemical composition also remained similar among treatments (Table 4). Moreover, muscle chemical composition reflected synthetic vitamin E inclusion, with muscle from fish fed VITE diet showing significantly higher α-tocopherol values than all remaining diets (13.7 mg kg w w^−1^; Table 4).

In fish plasma, innate immune parameters and bactericidal activity did not display any significant differences between dietary treatments (Table 5).

### 3.3. Effects on Liver Oxidative Stress Parameters

Glutathione reductase (GR) activity was significantly higher in CO and CTRL when compared to TO (Table 6). Lipid peroxidation (LPO) in fish fed the experimental diets was similar to CTRL; however, CO was significantly higher when compared to VITE. The activity of the remaining antioxidant enzymes, as well as the glutathione content and total antioxidant capacity (TAC), showed no significant differences between treatments.

### 3.4. Effects on Antioxidant Potential and Lipid Oxidation of Muscle

Despite the fact that muscle from fish fed VITE had five times the amount of α-tocopherol compared to the remaining diets, a two-way ANOVA comparison did not show any discernible effects on muscle antioxidant potential (Table 7). No statistical differences between dietary treatments were found for lipid peroxidation, DPPH^•^ and ABTS^•+^ in European sea bass muscle. Likewise, no statistical differences could also be perceived in either lipid peroxidation or ABTS^•+^ between Day 0 (fish muscle sampled immediately after the feeding trial) and Day 8 (muscle sampled after 8 days storage on ice). However, DPPH^•^ showed differences between Day 0 and Day 8, with higher values after 8 days on ice.

### 3.5. Effects on Sea Bass Skin, Fillet Colour and Muscle Texture

The effects of the experimental diets on European sea bass skin and fillet colour and textural properties were evaluated, both before and after 8 days on ice (Table 7). The two-way ANOVA showed no statistical differences in skin colour between dietary treatments. However, storage time induced skin colour changes, namely a decrease in yellowness (b*), chroma (C*), as well as an increase in hue angle (h*). Compared to CTRL, muscle from fish fed with the vegetable coproduct diets showed lower b* values, while fillets from fish fed with CO showed lower C*, whilst those fed with CA showed higher h*. After storage time, fish muscle showed higher lightness (L*) and h*, as well as lower b* and C* values. A two-way ANOVA analysis revealed that all muscle textural parameters suffered considerable alterations between the first and last day of storage. However, gumminess seemed to be the only parameter modulated by the experimental diets. Namely, CO displayed higher levels of gumminess when compared to TO. However, no experimental diets displayed any significant differences compared to CTRL.

### 3.6. Consumer Acceptance of Sea Bass Fillets

Consumers’ overall liking of fish samples were generally high (>7.5 out of 9) and without significant differences between treatments (Table 8).

**Figure 2:**
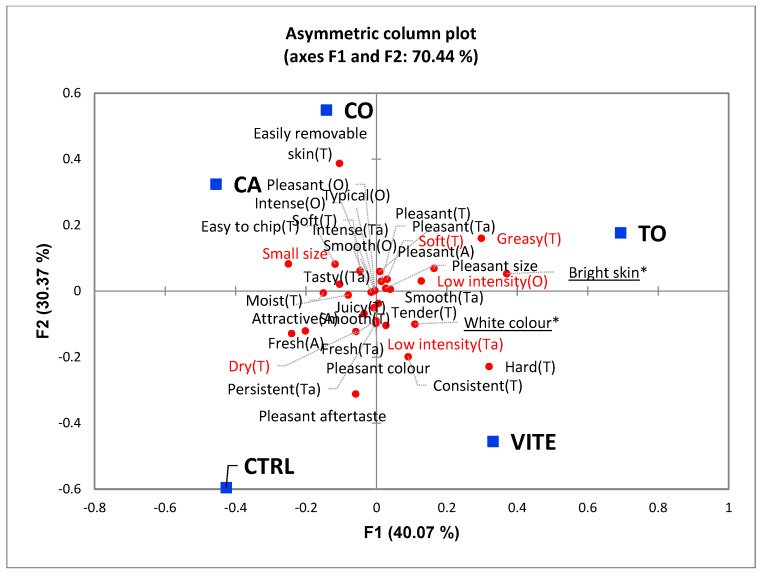
Correspondence analysis applied to open comments data regarding the evaluation of European sea bass fillet by consumers (n = 60). Samples from the same part of the fish muscle (5) were presented, each corresponding to a different dietary treatment. Attributes in red were mentioned as negative aspects; attributes in black were mentioned as positive aspects. (*) underlined attributes with a significant difference between samples, at a significance level of 5%.

Correspondence analysis applied to open comments data (positive and negative) regarding the evaluation of European sea bass (Figure 2) highlights the similarities and dissimilarities perceived by consumers for fillets from different diets, allowing a relevant and more generalized overview of the results. This biplot configuration explains 70.4% of total variance of the experimental data. Fillets from fish fed the CTRL were mainly associated with freshness, pleasant and attractive appearance, and a persistent and pleasant aftertaste, while the VITE was associated with a white colour, consistent and hard texture, and negatively correlated with dry texture. Moreover, fillets from fish fed diets CA and CO were mostly associated with terms related with odour such as pleasant, intense and typical, and also easily removable skin (more on the CO sample) and easy to chip. The TO sample was related with pleasant attributes regarding size, appearance (bright skin), texture and taste, but related with the negative attributes soft and greasy texture and low intense odour.

## 4. Discussion

Both synthetic and natural antioxidants are commonly included in aquafeeds in order to ameliorate the negative effects of oxidative stress, potentially increasing fish health and delaying flesh oxidation during storage [4], the physical and chemical conditions that occur during feed formulation, namely light exposure and high temperatures, may negatively affect antioxidant stability [76,77].

The high vulnerability of carotenoids to high temperatures is thoroughly documented [78]. Despite the fact that dried carrot and coriander used in this study revealed the presence of α-carotene and β-carotene, results showed a complete absence of these carotenoids in their respective experimental diets. In the case of lutein, although dried coriander was a very rich source, its 2% inclusion in CO diet was not reflected in the final dietary level. The measured amounts of lutein in the CO diet were 1.7 g 100 g^−1^ of DM, representing 68% of the expected value, i.e., 2.5 g 100 g^−1^ of DM. This result points towards a possible carotenoid degradation during the extrusion (110 °C) and/or drying (60 °C) stages of diet manufacture. Of the three ingredients used in this study, only coriander contained β-cryptoxanthin, but all diets just evidenced trace amounts of this pigments, again suggestion degradation during processing. The CA diet contained the highest amount of total polyphenols, thus significantly differing from the CTRL, possibly due to the dietary inclusion of dried carrot.

Despite synthetic vitamin E being a commonly used antioxidant in aquaculture, there is little knowledge regarding supplementation rates for commercial-sized European sea bass (*Dicentrarchus labrax*). Overall, quantification of vitamin E in experimental diets used in this study showed tocopherol amounts that reflected the different supplementation levels, since the VITE diet had five times more α-tocopherol than the remaining diets. Moreover, the dried vegetables added at a 2% inclusion rate did not provide any further significant contribution of vitamin E to the dietary formulations. However, supplementation doses did not reach targeted levels (500 mg kg^−1^ in VITE and 100 mg kg^−1^ in all other diets) as values measured after feed manufacturing procedures of extrusion and drying (126 mg kg^−1^ of DM and 25–30 mg kg^−1^ of DM, respectively) indicated a 24–30% α-tocopherol retention after feed manufacturing. During the feed formulation process, namely during the extrusion and drying stages, tocopherol suffers considerable temperature-induced degradation, an effect which is thoroughly documented [79,80,81]. Vitamin stability during extrusion depends on several factors, namely raw material, mixing, conditioning, temperature, pressure, moisture, energy input and extruder mechanical features [82]. According to Morin et al. [83], the chemical nature of the extruded matrix, moisture and temperature levels account for a variation of 63 ± 28% of tocopherol retention. Riaz and Ali [82] showed that the pelleting and extrusion processes alone can account for a 25% loss of vitamin E at around 80–90 °C, whilst higher extrusion temperatures, above 100 °C, increases the sensitivity of this vitamin significantly [83]. According to Anderson and Sunderland [76], most vitamin E losses occur over the course of aquafeeds extrusion process, before drying procedures, and might negatively affect the antioxidant potential of the diets. Effective technologies (e.g., colder extrusion and softer drying procedures, microencapsulation) able to protect both pigments and natural antioxidants sources prior extrusion should be envisaged.

The VITE diet exhibited less DPPH^•^ scavenging capacity than the CO diet, despite showing no differences when compared to control. Moreover, several synergistic mechanisms between different natural antioxidants, such as those found in coriander, are known to heighten the antioxidant potential of biological samples, generating superior antioxidant characteristics as opposed the sums of each individual one [84,85,86]. When compared to the control, the antioxidant capacity measured in diet VITE does not seem dose responsive. Previous studies suggested that the DPPH and ABTS method can be employed to examine lipophilic antioxidants such as tocopherols (vitamin E) [85,87]. However, the DPPH^•^ reaction rates are highly influenced by solvent composition [88], suggesting that results have to be interpreted with caution. In the present study, the DPPH and ABTS assays were carried out using the same extract employed for polyphenol quantification (via the Folin–Ciocalteau method), that is, methanol and distilled water (4:1, vv^−1^). This mixture is optimal for polyphenols due to the solvent’s polarity [89], but methanol is not the most appropriate solvent for tocopherol extraction, as it is a hydrophilic substance [90,91]. Thus, observed values from the DPPH and ABTS assays might not reflect the totality of radical scavenging capacity resulting from the addition of synthetic vitamin E, which may explain the lack of differences between VITE and the CTRL diet.

The chemical structure of carotenoids and polyphenols allows these compounds to regulate antioxidant activity due to their radical-scavenging properties [92]. Specifically, the antioxidant activity of lutein [93,94], β-carotene [93,95], lycopene [93,96], α-carotene and β-cryptoxanthin [93] has been thoroughly evaluated in in vitro studies. Supplementation of fish feeds with carotenoids has shown health associated benefits, acting as antioxidants and immunostimulants, enhancing fish resistance to bacterial and fungal diseases [97]. Additionally, polyphenols show an ability of scavenging free radicals, assisting in hindering the negative effects of ROS such as singlet oxygen, peroxynitrite and hydrogen peroxide, which must be continually removed from cells to maintain healthy metabolic function [98,99]. Natural phenolic compounds can also serve as potential additives for preventing quality deterioration or to retain the quality of fish and fish products [100], and seem to be rather resistant to deactivation via high-temperature extrusion-cooking process [101]. This antioxidant potential of polyphenols in terms of direct and indirect inhibition or suppression of oxidation processes is thoroughly established, mostly through trolox equivalent antioxidant capacity assays, as well as their scavenging capacity regarding the stable free radical 2,2-diphenyl-1-picryl-hydrazyl, commonly known as DPPH, due to its affinity with fat-soluble hydrophobic compounds, as is the case with polyphenols [102,103]. In this study, the CA diet revealed higher amounts of phenolic compounds (1179.1 ± 103.4 mg GAE 100 g^−1^ DM) than all remaining diets. This could be advantageous for the conservation of feed properties, as the proven antioxidant potency of polyphenols [17,100] might provide additional resistance to feed oxidation during storage.

Data from this study show that the addition of natural antioxidants to the experimental diets did not affect any of the evaluated immune parameters in fish. Moreover, considering the results of liver antioxidant activity observed in this study, GR was significantly higher in fish fed CTRL and CO diets when compared to fish fed with the TO diet. GR is an essential enzyme for catalysing the reaction that reduces oxidized glutathione (GSSR) into reduced glutathione (GSH) [104], the sum of which comprises total glutathione content (TG). GSH is an essential cofactor for antiperoxidative enzymes such as glutathione peroxidase (GPx) [104]. Therefore, a lower GR associated with the TO diet, in combination with liver lipid peroxidation data that showed no significant differences between TO and CTRL, might mean that fish fed with TO require a lower endogenous antioxidant activity in order to maintain cell homeostasis. However, TAC, which is specific for non-enzymatic antioxidants, displayed no differences between experimental treatments, meaning that we cannot directly attribute this lower production of GR to an increase in antioxidant potential stemming from exogenous antioxidants. As expected, due to the absence of differences between treatments regarding TG, glutathione-dependent antiperoxidative enzymes GPx and GST also did not show any differences when experimental diets were compared to the CTRL, as these enzymes require glutathione as a cofactor in order to perform their biological functions [105]. The antioxidant function of GPx and GST is largely dependent on its interaction with TG [106], neutralizing hydroperoxides as GSH is oxidized to GSSG by GR. Considering the results obtained in this study, we can observe that a lesser production of GR did not lead to differences in glutathione dependent enzymes. Moreover, the heightened dose of polyphenols in the CA diet did not translate into additional antioxidant protection in fish liver. This raises further questions concerning the bioavailability of these polyphenols in sea bass organism. The bioaccessibility and bioavailability of natural antioxidants in the organism not only relies on the concentration of bioactive compounds in the ingredient, but also on dosage and form of administration, composition of the feed matrix, while also being heavily influenced by other factors such as pH variations, enzyme action and digestion time [107,108]. Hence, further research is still warranted to clarify the full potential of natural antioxidant sources for inclusion in aquafeeds.

Overall, neither a heightened dose of vitamin E compared to standard values nor a 2% inclusion of natural antioxidant induced an upregulation of sea bass liver antioxidant system. Evaluation of the antioxidant potential present in these diets after the manufacturing process (extrusion and drying) only provides a limited perspective on their potential biological effect, since this is also greatly conditioned by their digestive bioaccessibility and bioavailability as discussed above.

Dietary vitamin E and natural antioxidants at the levels used in this study had no effects on fish proximate composition and feed conversion rate, confirming data obtained by Gatta et al. [35].

The beneficial effects of vitamin E as an antioxidant have been thoroughly evaluated in teleost fish, namely gilthead seabream (*Sparus aurata*) [109], red sea bream (*Pragus major*) [110] and rainbow trout (*Oncorhynchus mykiss)* [111,112]. However, in Atlantic salmon (*Salmo salar*) (IBW = 64 g), high supplementation levels of up to 1100 mg kg^−1^ dietary vitamin E did not affect fish antioxidant defence, lipid peroxidation and overall fish muscle resistance to oxidative stress [113]. Indeed, the available literature shows that the effectiveness of vitamin E as an antioxidant is largely dependent on fish life stage and species [4].

Overall, studies concerning the antioxidant effects of vitamin E of commercial-sized European sea bass are rare. Silva et al. [114] recommended a dosage of 500 mg kg^−1^ of vitamin E for adult sea bass. However, data obtained in this study suggests that a 500 mg kg^−1^ inclusion of vitamin E (VITE diet) in European sea bass feeds have no beneficial effect on muscle antioxidant potential compared to the traditional dosage of 100 mg kg^−1^ (CTRL diet). Gatta et al. [35] also reported decreased lipid peroxidation rates in sea bass fillets when α-tocopherol supplementation was increased from 139 mg kg^−1^ feed to 493 mg kg^−1^, but this could not be confirmed in the present study. It should be noted that all these results were obtained in optimised non-stressful rearing conditions for sea bass. Different conclusions regarding the antioxidant potential of these diets might have been reached if fish were submitted to a stress challenge, as enzymatic responses to oxidative stress are particularly promoted when fish face a pro-oxidant challenge [115]. Further studies should hence be envisaged to explore fish response to stressful conditions after being fed natural antioxidant sources.

Although vegetable coproduct inclusion and different vitamin E inclusion levels failed to show any antioxidant benefits in European sea bass muscle, significant differences were identified between Day 0 and Day 8. Namely, the antioxidant potential measured through the scavenging potential for DPPH^•^ was higher in fish stored in ice for 8 days, irrespective of the dietary treatments. This might have contributed towards the absence of differences in muscle LPO between Day 0 and Day 8, showing that lipid oxidation levels were similar between samples from both days. Likewise, Gatta et al. [35] showed that vitamin E supplementations between 139 mg kg^−1^ and 942 mg kg^−1^ fed to European sea bass (IBW = 200 g) showed no differences in terms of muscle lipid peroxidation between the first and last days of a 12-day storage time. The ability to scavenge free radicals is essential for ameliorating the negative effects of oxidative stress. Due to its relative stability, the free radical DPPH^•^ is a prime candidate as a first approach for evaluation of radical scavenging activity [116]. The eventual breakdown of cohesiveness between tissues, liquefaction of most organs and subsequent decomposition of proteins by hydrolysis leads to an increase in amino acid content, which might consequentially increase muscle free radical scavenging activity [117]. Moreover, naturally-occurring ROS via interactions with coproducts generated through the natural functioning processes of electron transport chains [118], cease functioning after death. Thus, a post-mortem increase in amino acid content accompanied by a lesser formation of ROS might explain this increase in DPPH^•^ radical scavenging activity. Plant by-products have also been proven effective in delaying chemical changes and microbial growth, as well as maintaining sensory characteristics and extending the shelf-life of seafood during refrigerated storage [102]. Natural antioxidants, namely phenolic compounds can have positive effects in terms of upregulation of muscle antibacterial properties and retardation of bacterial growth [102], which would translate into a larger product shelf-life. This was, however, not accessed in the present study and merits further evaluation.

Besides their antioxidant properties, carotenoids are also sources of pigments, and their deposition in tissues may affect skin/muscle colour and appearance in fish [119]. In this study, instrumental colour data of fish muscle was consistent with data found in the literature [120]. Moreover, all diets with natural antioxidant inclusion showed a significant decrease in muscle yellowness (b*) when compared to CTRL, whilst fish fed with CA presented a significantly higher h* than CTRL. Dietary coriander (CO) was associated with decreased muscle chroma (C*). Despite the evidence for dietary carotenoid degradation, the different diets still seem to modulate fish muscle colour. However, these differences in raw muscle colorimetric analyses between TO, CA and CO, when compared to CTRL, could not be perceived in the cooked muscle slices that were equally well accepted by the sensory panel. Similarly, in large-sized European sea bass, significant alterations in fillet colour of fish fed with *Isochrysis* sp., could not be detected by a sensory panel [120]. It is important to note that consumers were only able to significantly differentiate two attributes. TO group presented brighter skin than others, while VITE samples presented a white colour significantly different from the other groups. Both differences were mentioned as positive aspects.

This is particularly important in commercial fish species, since colour and visual appearance are known to influence market value, flavour perception and acceptability of fish food products [121], thus providing a measurable parameter for flesh freshness and ultimately affecting consumer perception quality [122,123]. Although differences in food colour can exert considerable influence on taste and consumers’ perception [121], overall liking scores in terms of consumer acceptability, showed no significant differences between samples. Indeed, all samples had a high average score value, in line with a high mention of positive comments, revealing that consumers were unable to detect differences between dietary treatments.

One of the most important freshness quality attributes of fish muscle is texture, which is heavily dependent on several parameters such as hardness, cohesiveness, springiness, chewiness, resilience and adhesiveness, as well as fibre detachment and internal cross-linking of connective tissue [124]. In terms of muscle texture, there were no significant differences among dietary treatments. Although hardness, adhesiveness, cohesiveness, gumminess and chewiness decreased after storage time, accompanied by an increase in springiness and resilience, none of these parameters were affected by the experimental diets.

## 5. Conclusions

Vitamin E and carotenoid content of extruded diets showed signs of degradation during the feed manufacturing process. Dietary vitamin E and natural antioxidants at the levels used in this study had very limited effects on European sea bass growth or body composition, immunomodulatory response, muscle and liver antioxidant potential, organoleptic properties or consumer acceptance. Neither a heightened inclusion dose of 500 mg kg^−1^ of vitamin E, nor a 2% inclusion of natural antioxidants provided additional antioxidant protection, compared to fish fed diets with a regular dose of 100 mg kg^−1^ of vitamin E. It should be noted that all these results were obtained under optimised non-stressful rearing conditions for sea bass. A pro-oxidant challenge is recommended to fully ascertain the fish responsiveness towards the inclusion of antioxidants. Moreover, in order to protect pigments and natural antioxidants throughout the feed manufacturing process, further research into alternative technologies is of paramount importance to produce cost-effective functional diets.

## Figures and Tables

**Table 1:** Proximate composition, carotenoids, vitamin E and antioxidant potential of ingredients.

Ingredients			
	Tomato	Carrot	Coriander
**Proximate composition (% DM)**			
Dry matter (DM)	77.3 ± 1.3	83.3 ± 1.3	93.4 ± 1.6
Crude protein	16.6 ± 0.4	12.4 ± 0.2	38.3 ± 0.1
Crude fat	3.9 ± 0.01	0.9 ± 0.11	3.2 ± 0.12
Ash	10.4 ± 0.7	7.2 ± 0.04	18.1 ± 0.7
Gross Energy (kJ/g)	21.0 ± 0.03	18.3 ± 0.03	17.5 ± 0.01
**Main carotenoids (mg 100 g^−1^ DM) ^1^**			
α-carotene	BQL	4.7 ± 0.3	14.6 ± 0.6
β-carotene	BQL	BQL	57.3 ± 13.1
Lutein	1.1 ± 0.2	BQL	124.5 ± 2.2
Lycopene	18.3 ± 3.6	-	-
β -cryptoxanthin	-	-	0.6 ± 0.04
**Vitamin E (mg kg^−1^ DM)**			
α-tocopherol	32.6 ± 4 ^b^	10.5 ± 3.2 ^c^	48.3 ± 1.9 ^a^
**Phenolic content (mg of GAE per 100 g DM) ^2^**			
Total polyphenols	729.6 ± 20.6 ^b^	2478.7 ± 154.9	528.3 ± 27.6 ^c^
**Antioxidant potential (mg of GAE or TE per 100 g DM) ^2^**			
ABTS^•+^	62.0 ± 3.6 ^c^	104.9 ± 2.8 ^b^	115.2 ± 0.2 ^a^
DPPH**^•^**	21.0 ± 0.9 ^c^	47.2 ± 4.2 ^b^	74.2 ± 0.2 ^a^

Values are presented as mean ± standard deviation (n = 9). Different superscript letters within each row indicate significant differences (*p* < 0.05). ^1^ “BQL” stands for “below quantification limit”. The quantification limit for lutein was 5.9 × 10^−6^ mg per 100 g of DM, α-carotene was 1.3 × 10^−5^ mg per 100 g of DM and β-carotene was 1.1 × 10^−5^ mg per 100 g of DM. ^2^ Total polyphenols are expressed in g of gallic acid equivalents (GAE) per 100 g DM. ABTS^•+^ and DPPH^•^ are expressed in mg of TE per 100 g DM.

**Table 2:** Ingredients, proximate composition and antioxidant potential of the experimental diets.

Dietary Treatments ^1^					
	CTRL	VITE	TO	CA	CO
**Ingredients (% of feed)**					
Fishmeal super prime	10.0	10.0	10.0	10.0	10.0
Porcine blood meal	2.5	2.5	2.5	2.5	2.5
Poultry meal	5.0	5.0	5.0	5.0	5.0
Soy protein concentrate	16.0	16.0	16.0	16.0	16.0
Wheat gluten	9.5	9.5	9.5	9.5	9.5
Corn gluten	7.0	7.0	7.0	7.0	7.0
Soybean meal 48	10.0	10.0	10.0	10.0	10.0
Rapeseed meal	5.0	5.0	5.0	5.0	5.0
Wheat meal	16.5	16.4	14.5	14.5	14.5
Fish oil	5.1	5.1	5.1	5.1	5.1
Rapeseed oil	9.4	9.4	9.4	9.4	9.4
Soy lecithin	0.2	0.2	0.2	0.2	0.2
Vitamin and mineral Premix ^2^	0.2	0.2	0.2	0.2	0.2
Lutavit E50	0.0	0.08	0.0	0.0	0.0
Brewer’s yeast	2.5	2.5	2.5	2.5	2.5
MAP	0.9	0.9	0.9	0.9	0.9
DL-Methionine	0.2	0.2	0.2	0.2	0.2
Tomato	-	-	2.0	-	-
Carrot	-	-	-	2.0	-
Coriander	-	-	-	-	2.0
**Chemical composition (% DM or kJ g^−1^ DM)**					
Dry matter	91.2	92.3	95.8	93.2	95.8
Crude protein	49.3	49.4	49.5	49.5	49.8
Crude fat	18.2	18.2	18.2	17.9	18.3
Ash	5.0	5.0	5.1	5.1	5.3
Gross Energy	23.4	23.4	23.6	23.4	23.5
Phosphorous	0.9	0.9	0.8	0.9	0.8
**Main Carotenoids (mg 100 g** **^−1^ DM) ^3^**					
Lutein	1.5 ± 0.1 ^ab^	0.6 ± 0.2 ^c^	1.8 ± 0.2 ^a^	1.0 ± 0.1 ^bc^	1.7 ± 0.05 ^a^
β-cryptoxanthin	BQL	BQL	BQL	BQL	BQL
**Vitamin E (mg kg** **^−1^ of DM)**					
α-tocopherol	23.7 ± 1.4 ^b^	126.0 ± 18.3 ^a^	30.5 ± 3.9 ^b^	25.2 ± 0.3 ^b^	25.4 ± 3.3 ^b^
**Phenolic content (mg of GAE per 100 g DM) ^4^**					
Total polyphenols	513.6 ± 24.5 ^b^	459.7 ± 31.0 ^b^	537.9 ± 27.8 ^b^	1179.1 ± 103.4 ^a^	536.0 ± 26.1 ^b^
**Antioxidant potential (mg of TE per g DM) ^4^**					
DPPH^•^	14.3 ± 0.3 ^b^	13.7 ± 0.4 ^b^	11.4 ± 0.6 ^c^	10.6 ± 0.3 ^c^	16.6 ± 1.1 ^a^
ABTS^•+^	54.7 ± 1.5	50.3 ± 3.4	55.0 ± 1.2	54.5 ± 2.1	51.4 ± 1.8

Values are presented as mean ± standard deviation (n = 3) when necessary. Different superscript letters within each row indicate significant differences (*p* < 0.05). ^1^ CTRL, control, vegetable based-diet with 100 mg kg^−1^ of total vitamin E; VIT E, vitamin E, vegetable based-diet with 500 mg kg^−1^ of total vitamin E; CA, TO and CO, 2% of natural antioxidants, carrot, tomato and coriander, respectively, at the expense of wheat meal of CTRL diet. ^2^ Vitamins are expressed mg or IU per kg of diet: vitamin A (retinyl acetate), 20,000 IU; vitamin D3 (DL-cholecalciferol), 2000 IU; vitamin K3 (menadione sodium bisulfite), 25 mg; vitamin B1 (thiamine hydrochloride), 30 mg; vitamin B2 (riboflavin), 30 mg; vitamin B6 (pyridoxine hydrochloride), 20 mg; vitamin B12 (cyanocobalamin), 0.1 mg; vitamin B5 (pantothenic acid), 100 mg; vitamin B3 (nicotinic acid), 200 mg; vitamin B9 (folic acid), 15 mg; vitamin H (biotin), 3 mg; betaine, 500 mg; inositol, 500 mg; choline chloride, 1000 mg; vitamin C (stay C), 1000 mg; vitamin E, 100 mg. Minerals (% or mg/kg diet): Mn (manganese oxide), 9.6 mg; I (potassium iodide), 0.5 mg; Cu (cupric sulphate), 9 mg; Co (cobalt sulphate), 0.65 mg; Zn (zinc oxide), 7.5 mg; Se (sodium selenite), 0.01 mg; Fe (iron sulphate), 6 mg; Cl (sodium chloride), 2.41%; Ca (calcium carbonate), 18.6%; NaCl (sodium), 4%. ^3^ “BQL” stands for “below quantification limit”. The quantification limit for lutein was 5.9 × 10^−6^ g per 100 g of DM and 1.3 × 10^−12^ for β-cryptoxanthin. ^4^ Total polyphenols are expressed in mg of gallic acid equivalents (GAE) per 100 g DM. ABTS^•+^ and DPPH^•^ are expressed in mg of TE per 100 g DM.

**Table 3:** Growth and whole-body composition of *Dicentrarchus labrax* fed with the experimental diets for 87 days.

Dietary Treatments ^1^						
	CTRL	VITE	TO	CA	CO	*p*-Value
**Growth**						
Final body weight	239.0 ± 14.3	245.7 ± 19.0	244.8 ± 1.1	247.7 ± 6.4	236.7 ± 18.2	0.8
Final length	27.3 ± 0.1	27.4 ± 0.6	27.5 ± 0.1	27.3 ± 0.2	27.1 ± 0.4	0.7
K	1.2 ± 0.1	1.2 ± 0.01	1.2 ± 0.01	1.2 ± 0.01	1.2 ± 0.04	0.1
VFI	1.0 ± 0.1	0.9 ± 0.1	1.0 ± 0.1	0.9 ± 0.1	1.0 ± 0.1	0.8
DGI	1.6 ± 0.1	1.6 ± 0.2	1.6 ± 0.02	1.6 ± 0.1	1.5 ± 0.2	0.8
FCR	1.2 ± 0.1	1.1 ± 0.1	1.2 ± 0.1	1.1 ± 0.04	1.2 ± 0.04	0.3
PER	1.7 ± 0.1	1.8 ± 0.1	1.7 ± 0.1	1.8 ± 0.1	1.7 ± 0.1	0.3
**Whole Body Composition (% wet weight, ww^−1^)**						
Moisture	62.3 ± 0.7	62.5 ± 0.9	63.4 ± 0.9	62.1 ± 1.6	62.4 ± 1.2	0.7
Ash	3.5 ± 0.2	3.6 ± 0.3	4.1 ± 0.6	3.5 ± 0.1	4.0 ± 0.1	0.1
Protein	19.2 ± 0.8	19.2 ± 0.1	18.3 ± 0.5	18.7 ± 0.5	18.6 ± 0.5	0.2
Lipids	15.8 ± 1.3	15.9 ± 0.9	15.0 ± 0.8	16.4 ± 2.3	15.6 ± 1.2	0.8
Energy (kJ/g)	9.9 ± 0.3	10.1 ± 0.3	9.7 ± 0.1	10.0 ± 0.8	9.8 ± 0.4	0.8
Phosphorus	0.6 ± 0.04	0.6 ± 0.05	0.7 ± 0.1	0.6 ± 0.03	0.8 ± 0.1	0.1
**Gain (g or kj/ABW kg/day)**						
Dry matter	3.2 ± 0.3	3.2 ± 0.4	3.1 ± 0.2	3.3 ± 0.4	3.1 ± 0.5	0.9
Protein	1.6 ± 0.01	1.7 ± 0.1	1.5 ± 0.1	1.6 ± 0.04	1.5 ± 0.1	0.2
Lipids	1.5 ± 0.3	1.5 ± 0.2	1.4 ± 0.1	1.6 ± 0.4	1.4 ± 0.3	0.9
Energy	88.3 ± 9.0	94.6 ± 8.8	88.3 ± 1.8	93.6 ± 15.8	86.5 ± 13.1	0.8
Phosphorus	0.03 ± 0.002	0.04 ± 0.01	0.05 ± 0.01	0.03 ± 0.003	0.05 ± 0.03	0.2

For all the items n = 3, except for initial and final body weight, which had n = 60.

**Table 4:** Somatic indexes and chemical composition of muscle and liver of *Dicentrarchus labrax* fed with the experimental diets for 87 days.

	Dietary Treatments					
	CTRL	VITE	TO	CA	CO	*p*-Value
**Somatic Indexes**						
Hepatosomatic index	2.2 ± 0.7	2.1 ± 0.5	2.1 ± 0.3	2.1 ± 0.3	2.4 ± 0.8	0.8
Viscerosomatic index	6.1 ± 0.8	6.5 ± 1.1	6.6 ± 0.6	6.4 ± 1.1	5.8 ± 0.7	0.2
**Muscle chemical composition ^1^**						
Moisture	73.7 ± 1.9	72.9 ± 2.3	72.6 ± 0.5	73.5 ± 1.9	73.9 ± 1.0	0.9
Ash	1.4 ± 0.1	1.4 ± 0.01	1.5 ± 0.05	1.5 ± 0.1	1.4 ± 0.03	0.4
Lipid	4.4 ± 1.1	5.8 ± 1.8	5.1 ± 0.2	4.3 ± 1.9	4.2 ± 1.1	0.6
α-tocopherol	5.1 ± 2.2 ^b^	13.7 ± 3.2 ^a^	5.6 ± 1.6 ^b^	4.2 ± 1.1 ^b^	3.9 ± 0.7 ^b^	**<0.001**
**Liver chemical composition ^1^**						
Moisture	42.4 ± 2.3	46.3 ± 2.0	42.2 ± 1.8	42.5 ± 1.4	42.8 ± 6.3	0.5
Ash	0.8 ± 0.2	0.8 ± 0.03	0.8 ± 0.05	0.9 ± 0.1	0.9 ± 0.1	0.7
Lipid	35.1 ± 8.7	28.9 ± 4.6	33.2 ± 2.7	33.6 ± 4.7	31.2 ± 3.7	0.7

Values are presented as mean ± standard deviation (n = 12). Different superscript letters within each row indicate significant differences (*p* < 0.05). ^1^ Moisture, lipid and ash are presented in % of ww^−1^. α-tocopherol is presented in mg kg ww^−1^.

**Table 5:** Innate immune parameters of *Dicentrarchus labrax* evaluated after 87 days of feeding the experimental diets.

	Dietary Treatments					
	CTRL	VITE	TO	CA	CO	*p*-Value
Lysozyme ^1^	822.7 ± 45.9	741.2 ± 48.6	893.3 ± 61.2	865.0 ± 42.9	868.5 ± 65.1	0.3
Peroxidase ^1^	617.1 ± 102.0	491.3 ± 77.9	529.5 ± 76.7	523.5 ± 97.5	496.0 ± 93.5	0.9
ACH50 ^1^	336.1 ± 21.9	331.7 ± 23.4	302.9 ± 18.6	345.4 ± 23.6	313.8 ± 17.2	0.6
IgM ^1^	0.2 ± 0.1	0.1 ± 0.1	0.2 ± 0.1	0.2 ± 0.1	0.2 ± 0.1	0.4
Pdp activity ^2^	9.5 ± 10.5	13.6 ± 9.6	19.9 ± 13.0	17.1 ± 10.2	5.6 ± 9.6	0.1
VA activity ^2^	26.8 ± 8.4	22.1 ± 9.1	26.0 ± 9.0	28.5 ± 8.6	20.8 ± 12.6	0.5

Values are presented as mean ± standard error (n = 12). ^1^ Lysozyme, peroxidase, ACH50 are presented in EU mL^−1^; IgM is presented in OD 450 nm. ^2^ Plasma bactericidal activity of European sea bass fed different dietary treatments against *Vibrio anguillarum* (VA) and *Photobacterium damselae* subsp. *piscicida* (Pdp). Values are present in % of non-viable bacteria. One-way ANOVA was used to test differences. No significant differences observed between different diets (*p* > 0.05).

**Table 6:** Enzymatic/non-enzymatic parameters in *Dicentrarchus labrax* liver.

Dietary Treatments						
	CTRL	VITE	CA	CO	TO	*p*-Value
**Enzymatic parameters**						
CAT	33.5 ± 19.4	43.9 ± 11.3	35.1 ± 17.7	41.2 ± 19.7	32.9 ± 11.4	0.4
GST	158.7 ± 65.4	155.8 ± 64.9	170.3 ± 60.3	232.9 ± 76.3	186.2 ± 82.7	0.1
GR	3.8 ± 1.6 ^a^	3.5 ± 1.0 ^ab^	3.6 ± 1.5 ^ab^	4.1 ± 0.9 ^a^	2.3 ± 1.0 ^b^	**0.01**
TG	0.8 ± 0.4	0.8 ± 0.3	1.0 ± 0.4	1.1 ± 0.4	0.7 ± 0.2	0.05
GPx	2.1 ± 0.9	1.6 ± 0.7	1.7 ± 1.1	2.24± 0.6	1.5 ± 0.6	0.2
**Non-enzymatic parameters**						
LPO	96.4 ± 18.2 ^ab^	92.1 ± 34.3 ^b^	109.0± 36.2 ^ab^	147.7 ± 55.6 ^a^	114.6± 48.4 ^ab^	**0.04**
TAC	0.03 ± 0.02	0.03 ± 0.01	0.02 ± 0.01	0.03 ± 0.01	0.03 ± 0.01	0.8

Different superscript letters indicate within each row differences between diets (*p* < 0.05). Glutathione s-transferase (GST), glutathione reductase (GR), glutathione peroxidase (GPx) and total glutathione (TG) are in nmol min^−1^ mg^−1^ protein, catalase (CAT) is in μmol min^−1^ mg^−1^ lipid peroxidation (LPO) is in nmol MDA g liver^−1^. Total antioxidant capacity (TAC) is calculated in nmol mg tissue^−1^. Values are presented as mean ± standard deviation (n = 12) per dietary treatment.

**Table 7:** Antioxidant potential, skin and muscle colour and texture of *Dicentrarchus labrax* in the first sampling day (Day 0) and after 8 days of storage (Day 8).

Dietary Treatments ^1^													
	Day 0					Day 8					*p*-Value		
	CTRL	VITE	TO	CA	CO	CTRL	VITE	TO	CA	CO	Diet	Day	D × D
**Muscle antioxidant potential**													
DPPH^•^	0.2 ± 0.04	0.1 ± 0.04	0.2 ± 0.05	0.1 ± 0.04	0.1 ± 0.04	0.2 ± 0.1 *	0.2 ± 0.04 *	0.2 ± 0.1 *	0.3 ± 0.1 *	0.3 ± 0.1 *	0.9	**<0.01**	0.5
ABTS^•+^	0.6 ± 0.1	0.6 ± 0.1	0.6 ± 0.1	0.6 ± 0.1	0.6 ± 0.1	0.6 ± 0.1	0.6 ± 0.1	0.6 ± 0.05	0.6 ± 0.1	0.6 ± 0.1	0.9	0.9	0.5
LPO	33.8 ± 3.0	35.3 ± 5.1	35.2 ± 3.4	36.8 ± 6.3	36.5 ± 4.6	34.0 ± 2.7	32.1 ± 2.4	33.9 ± 3.4	31.5 ± 1.7	33.4 ± 2.8	0.4	0.4	0.5
**Colour of skin**													
L^*^	53.7 ± 5.2	53.6 ± 3.4	52.6 ± 4.6	55.3 ± 4	50.2 ± 5.3	52.7 ± 4.6	51.1 ± 3.5	50.8 ± 3.3	50.3 ± 4.0	55.4 ± 2.1	0.7	0.2	0.1
a*	−4.3 ± 0.5	−4.6 ± 0.4	−4.3 ± 0.7	−4.8 ± 0.7	−4.5 ± 0.4	−4.6 ± 0.7	−4.9 ± 0.4	−4.5 ± 0.7	−4.7 ± 0.4	−4.5 ± 0.3	0.1	0.1	0.4
b*	7.3 ± 1.9 *	7.4 ± 0.9 *	7.0 ± 1.4 *	8.1 ± 2.8 *	6.7 ± 1.0 *	4.7 ± 2.5	5.7 ± 1.4	5.3 ± 1.8	5.5 ± 0.7	5.3 ± 0.9	0.4	**<0.01**	0.6
C*	8.5 ± 1.7 *	8.7 ± 0.9 *	8.3 ± 1.3 *	9.5 ± 1.0 *	8.1 ± 0.9 *	6.9 ± 1.3	7.6 ± 1.1	7.1 ± 1.4	7.3 ± 0.2	7.0 ± 0.8	0.2	**<0.01**	0.6
h*	121.5 ± 5.6 *	122 ± 3.5 *	122.2 ± 5.9 *	124.3 ± 3.5 *	124.3 ± 3.5 *	137.6 ± 20.4	132.6 ± 7.8	132.5 ± 10.3	131.6 ± 6.4	130.9 ± 5.4	0.8	**<0.01**	0.5
**Colour of muscle**													
L*	42.8 ± 2.5 *	42.3 ± 2.0 *	42.2 ± 1.8 *	41.5 ± 1.3 *	41.5 ± 1.9 *	43.9 ± 2.28	44.2 ± 1.7	44.1 ± 3.4	43.9 ± 1.6	42.3 ± 2.0	0.1	**<0.01**	0.6
a*	−2.9 ± 0.5	−3.0 ± 0.6	−2.8 ± 0.4	−2.9 ± 0.5	−2.7 ± 0.5	−2.7 ± 0.72	−3.0 ± 0.7	−2.7 ± 0.7	−2.8 ± 0.7	−2.4 ± 0.7	0.2	0.2	0.9
b*	4.3 ± 1.1 ^a^*	3.9 ± 0.8 ^ab^*	3.7 ± 1.1 ^b^*	3.9 ± 0.7 ^b^*	3.6 ± 0.7 ^b^*	1.4 ± 0.67 ^a^	0.6 ± 0.6 ^ab^	0.7 ± 1.2 ^b^	0.5 ± 0.5 ^b^	0.5 ± 0.8 ^b^	**0.01**	**<0.01**	0.8
C*	5.3 ± 0.9 ^a^*	4.9 ± 0.6 ^ab^*	4.7 ± 0.9 ^ab^*	4.8 ± 0.7 ^ab^*	4.6 ± 0.6 ^b^*	3.2 ± 0.45 ^a^	3.1 ± 0.5 ^ab^	3.2 ± 0.5 ^ab^	2.9 ± 0.6 ^ab^	4.6 ± 0.6 ^b^	**0.02**	**<0.01**	0.7
h*	125.0 ± 10.3 ^b^*	128.9 ± 8.6 ^ab^*	128.7 ± 13.2 ^ab^*	127.0 ± 5.4 ^a^*	127.4 ± 7.6 ^ab^*	151.3 ± 16.2 ^b^	167.7 ± 13.1 ^ab^	164.9 ± 23.9 ^ab^	171.0± 11.3 ^a^	170.4 ± 15.7 ^ab^	**0.03**	**<0.01**	0.2
**Muscle Texture**													
Hardness	9.8 ± 2.0 *	8.0 ± 1.6 *	9.7 ± 1.8 *	9.6 ± 1.5 *	10.0 ± 1.4 *	3.3 ± 2.5	4.2 ± 2.9	2.8 ± 1.9	4.8 ± 2.7	4.7 ± 2.3	0.2	**<0.01**	0.1
Adhesiveness	−0.4 ± 0.1 *	−0.2 ± 0.1 *	−0.4 ± 0.2 *	−0.3 ± 0.1 *	−0.3 ± 0.1 *	−1.78 ± 0.8	−1.9 ± 0.6	−2.5 ± 0.6	−1.9 ± 0.9	−2.3 ± 0.6	0.05	**<0.01**	0.1
Springiness	1.1 ± 0.1 *	1.2 ± 0.2 *	1.11 ± 0.1 *	1.2 ± 0.2 *	1.2 ± 0.2 *	1.24 ± 0.25	1.2 ± 0.1	1.3 ± 0.28	1.31 ± 0.3	1.4 ± 0.3	0.5	**<0.01**	0.4
Cohesiveness	0.4 ± 0.04 *	0.4 ± 0.03 *	0.4 ± 0.1 *	0.4 ± 0.1 *	0.4 ± 0.1 *	0.3 ± 0.04	0.3 ± 0.1	0.3 ± 0.03	0.32 ± 0.03	0.31 ± 0.03	0.3	**<0.01**	0.4
Gumminess	3.5 ± 0.5 ^ab^*	3.1 ± 0.6 ^ab^	3.4 ± 0.5 ^b^*	3.7 ± 0.7 ^ab^*	4.0 ± 0.9 ^a^*	1.0 ± 0.8 ^ab^	1.3 ± 0.9 ^ab^	0.8 ± 0.6 ^b^	1.6 ± 0.9 ^ab^	1.5 ± 0.8 ^a^	**<0.01**	**<0.01**	0.4
Chewiness	4.2 ± 0.9 *	3.7 ± 1.2 *	3.9 ± 0.8 *	4.0 ± 0.8 *	4.2 ± 1.8 *	1.3 ± 1.0	1.6 ± 1.1	1.1 ± 0.7	1.9 ± 1.1	2.1 ± 1.1	0.1	**<0.01**	0.5
Resilience	0.6 ± 0.3 *	0.4 ± 0.1 *	0.5 ± 0.1 *	0.4 ± 0.1 *	0.4 ± 0.1 *	0.8 ± 0.5	0.8 ± 0.3	1.0 ± 0.6	0.9 ± 0.6	1.0 ± 0.5	0.8	**<0.01**	0.2

Values are presented as mean ± standard deviation (n = 12). Within a row, superscripted lowercase letters (^ab^) mean significant differences between diets, while (*) means differences between days (*p* < 0.05). Hardness and gumminess are in newtons, adhesiveness and chewiness are in joules. Muscle lipid peroxidation is in nmol MDA g liver^−1^, while DPPH/ABTS assays are in µmol TE mg^−1^ ww^−1^.

**Table 8:** Overall liking of fish samples with different dietary treatments.

Dietary Treatments	Mean ± SD
CTRL	7.5 ± 1.2
VITE	7.5 ± 1.5
TO	7.7 ± 1.0
CA	7.5 ± 1.0
CO	7.6 ± 1.1
*p*-value ^1^	0.651

Consumer acceptance (n = 60) of sea bass fillets using a 9-point hedonic scale. Five samples from the same part of the fish (slices from the anterior, middle or posterior part of the fish body) were presented, each corresponding to a different dietary treatment. ^1^ For the dietary treatment effect on a mixed-model three-way ANOVA.

## Data Availability

Data is contained within the article.

## References

[1] Tort L. (2011). Stress and immune modulation in fish. Dev. Comp. Immunol..

[2] Halliwell B., Gutteridge J.M.C. (2015). Free Radicals in Biology and Medicine.

[3] Baiano A., Del Nobile M.A. (2016). Antioxidant Compounds from Vegetable Matrices: Biosynthesis, Occurrence, and Extraction Systems. Crit. Rev. Food Sci. Nutr..

[4] NRC (2011). National Research Council (NRC): Nutrient Requirements of Fish and Shrimp.

[5] Taşbozan O., Gokce M., Catala A. (2017). Fatty Acids in Fish. Fatty Acids.

[6] Dominguez R., Pateiro M., Gagaoua M., Barba F.J., Zhang W., Lorenzo J.M. (2019). A Comprehensive Review on Lipid Oxidation in Meat and Meat Products. Antioxidants.

[7] FAO (2020). The State of World Fisheries and Aquaculture 2020–Sustainability in Action.

[8] Olmos-Soto J., Kim S.W. (2015). Functional Feeds in Aquaculture. Handbook of Marine Biotechnology.

[9] Encarnação P., Nates S.F. (2016). Functional feed additives in aquaculture feeds. Aquafeed Formulation.

[10] Aklakur M. (2018). Natural antioxidants from sea: A potential industrial perspective in aquafeed formulation. Rev. Aquac..

[11] Bharathi S., Cheryl A., Rajagopalasamy C., Uma A., Ahilan B., Aanand S. (2019). Functional feed additives used in fish feeds. Int. J. Fish. Aquat. Stud..

[12] European Comission (2017). Suspending the Authorisation of Ethoxyquin as a Feed Additive for All Animal Species and Categories.

[13] European Comission (2004). List of the Authorised Additives in Feedingstuffs Published in Application of Article 9t (b) of Council Directive 70/524/EEC Concerning Additives in Feedingstuffs (Text with EEA Relevance).

[14] Lanigan R.S., Yamarik T.A. (2002). Final report on the safety assessment of BHT. Int. J. Toxicol..

[15] Kulawik P., Fatih O., Glew R.H. (2013). Quality Properties, Fatty Acids, and Biogenic Amines Profile of Fresh Tilapia Stored in Ice. J. Food Sci..

[16] Reverter M., Bontemps N., Lecchini D., Banaigs B., Sasal P. (2014). Use of plant extracts in fish aquaculture as an alternative to chemotherapy: Current status and future perspectives. Aquaculture.

[17] Pezeshk S., Alishahi A. (2015). Effect of Plant Antioxidant and Antimicrobial Compounds on the Shelf-life of Seafood-A Review. Czech J. Food Sci..

[18] Elseady Y., Zahran E. (2013). Ameliorating effect of β-carotene on antioxidant response and hematological parameters of mercuric chloride toxicity in Nile tilapia (Oreochromis niloticus). Fish Physiol. Biochem..

[19] Bai S.C., Katya K., Yun H., Davis D.A. (2015). Additives in aquafeed: An overview. Feed and Feeding Practices in Aquaculture.

[20] Kousoulaki K., Sæther B.-S., Albrektsen S., Noble C. (2015). Review on European sea bass (Dicentrarchus labrax, Linnaeus, 1758) nutrition and feed management: A practical guide for optimizing feed formulation and farming protocols. Aquacult. Nutr..

[21] Ehsani A., Jasour M.S., Agh N., Hashemi M., Khodadadi M. (2018). Rancidity development of refrigerated rainbow trout (*Oncorhynchus mykiss*) fillets: Comparative effects of in vivo and in vitro lycopene. J. Sci. Food Agric..

[22] Sun T., Simon P.W., Tanumihardjo S.A. (2009). Antioxidant Phytochemicals and Antioxidant Capacity of Biofortified Carrots (Daucus carota L.) of Various Colors. J. Agric. Food Chem..

[23] Laribi B., Kouki K., M’Hamdi M., Bettaieb T. (2015). Coriander (Coriandrum sativum L.) and its bioactive constituents. Fitoterapia.

[24] Martí R., Roselló S., Cebolla-Cornejo J. (2016). Tomato as a Source of Carotenoids and Polyphenols Targeted to Cancer Prevention. Cancers.

[25] Udo I., Afia O. (2013). Optimization of Dietary Vitamin E (Tocopherols) in Fish: A Review. J. Agric. Food Environ..

[26] Sen C.K., Khanna S., Roy S. (2006). Tocotrienols: Vitamin E beyond tocopherols. Life Sci..

[27] Traber M.G., Atkinson J. (2007). Vitamin E, antioxidant and nothing more. Free Radic. Biol. Med..

[28] Blaner W.S. (2013). Vitamin E: The enigmatic one!. J. Lipid Res..

[29] Halver J.E., Halver J.E., Hardy R.W. (2003). The Vitamins. Fish Nutrition.

[30] Aggarwal B.B., Sundaram C., Prasad S., Kannappan R. (2010). Tocotrienols, the vitamin E of the 21st century: Its potential against cancer and other chronic diseases. Biochem. Pharmacol..

[31] Cuesta A., Esteban M.A., Ortuño J., Meseguer J. (2001). Vitamin E increases natural cytotoxic activity in seabream (*Sparus aurata* L.). Fish Shellfish Immunol..

[32] Lu Y., Liang X.-P., Jin M., Sun P., Ma H.-N., Yuan Y., Zhou Q.-C. (2016). Effects of dietary vitamin E on the growth performance, antioxidant status and innate immune response in juvenile yellow catfish (*Pelteobagrus fulvidraco*). Aquaculture.

[33] Kamireddy N., Jittinandana S., Kenney P., Slider S., Kiser R., Mazik P.M., Hankins J.A. (2011). Effect of Dietary Vitamin E Supplementation and Refrigerated Storage on Quality of Rainbow Trout Fillets. J. Food Sci..

[34] Ruff N., FitzGerald R.D., Cross T.F., Hamre K., Kerry J.P. (2003). The effect of dietary vitamin E and C level on market-size turbot (*Scophthalmus maximus*) fillet quality. Aquacult. Nutr..

[35] Gatta P.P., Pirini M., Testi S., Vignola G., Monetti P.G. (2000). The influence of different levels of dietary vitamin E on sea bass Dicentrarchus labrax flesh quality. Aquacult. Nutr..

[36] Guerriero G., Ferro R., Russo G.L., Ciarcia G. (2004). Vitamin E in early stages of sea bass (Dicentrarchus labrax) development. Comp. Biochem. Physiol.–A Mol. Integr. Physiol..

[37] Chakraborty S.B., Hancz C. (2011). Application of phytochemicals as immunostimulant, antipathogenic and antistress agents in finfish culture. Rev. Aquac..

[38] Sallam A.E., Mansour A.T., Srour T.M., Goda A.M.A. (2017). Effects of different carotenoid supplementation sources with or without sodium taurocholate on growth, feed utilization, carotenoid content and antioxidant status in fry of the European seabass, Dicentrarchus labrax. Aquacult. Res..

[39] Sallam A., Goda A., Sorur T. (2018). Evaluation of Natural and Synthetic Carotenoid Supplementation on Growth, Survival, Total Carotenoid Content, Fatty Acids Profile and Stress Resistance of European Seabass, *Dicentrarchus labrax*, Fry. Aquac. Stud..

[40] Peixoto M.J., Salas-Leitón E., Pereira L.F., Queiroz A., Magalhães F., Pereira R., Abreu H., Reis P.A., Gonçalves J.F.M., de Almeida Ozório R.O. (2016). Role of dietary seaweed supplementation on growth performance, digestive capacity and immune and stress responsiveness in European seabass (*Dicentrarchus labrax*). Aquac. Rep..

[41] Leser S. (2013). The 2013 FAO report on dietary protein quality evaluation in human nutrition: Recommendations and implications. Nutr. Bull..

[42] Porter S.D., Reay D.S., Bomberg E., Higgins P. (2018). Avoidable food losses and associated production-phase greenhouse gas emissions arising from application of cosmetic standards to fresh fruit and vegetables in Europe and the UK. J. Clean. Prod..

[43] Shalaby A.M., Khattab Y.A., Abdel Rahman A.M. (2006). Effects of Garlic (*Allium sativum*) and chloramphenicol on growth performance, physiological parameters and survival of Nile tilapia (*Oreochromis niloticus*). J. Venom. Anim. Tox. Incl. Trop. Dis..

[44] Pavaraj M., Balasubramanian V., Baskaran S., Ramasamy P. (2011). Development of immunity by extract of medicinal plant Ocimum sanctum on common carp *Cyprinus carpio* (L.). J. Immunol. Res..

[45] Takaoka O., Ji S.-C., Ishimaru K., Lee S.-W., Jeong G.-S., Ito J., Biswas A., Takii K. (2011). Effect of rotifer enrichment with herbal extracts on growth and resistance of red sea bream, Pagrus major (Temminck & Schlegel) larvae against Vibrio anguillarum. Aquacult. Res..

[46] Harikrishnan R., Balasundaram C., Jawahar S., Heo M.-S. (2012). Immunomodulatory effect of Withania somnifera supplementation diet in the giant freshwater prawn Macrobrachium rosenbergii (de Man) against Aeromonas hydrophila. Fish Shellfish Immunol..

[47] Sanches-Silva A., Costa D., Albuquerque T.G., Buonocore G.G., Ramos F., Castilho M.C., Machado A.V., Costa H.S. (2014). Trends in the use of natural antioxidants in active food packaging: A review. Food Addit. Contam. Part A Chem. Anal. Control Expo Risk Assess..

[48] Blancheton J.P. (2000). Developments in recirculation systems for Mediterranean fish species. Aquacult. Eng..

[49] AOAC (2006). Official Methods of Analysis of AOAC International.

[50] Folch J., Lees M., Sloane Stanley G.H. (1957). A simple method for the isolation and purification of total lipides from animal tissues. J. Biol. Chem..

[51] ISO (1996). Meat and Meat Products—Determination of Total Phosphorus Content—Spectrometric Method.

[52] Slavin M., Yu L.L. (2012). A single extraction and HPLC procedure for simultaneous analysis of phytosterols, tocopherols and lutein in soybeans. Food Chem..

[53] Gómez-García R., Campos D.A., Oliveira A., Aguilar C.N., Madureira A.R., Pintado M. (2021). A chemical valorisation of melon peels towards functional food ingredients: Bioactives profile and antioxidant properties. Food Chem..

[54] Xie P.J., Huang L.X., Zhang C.H., Zhang Y.L. (2015). Phenolic compositions, and antioxidant performance of olive leaf and fruit (*Olea europaea* L.) extracts and their structure–activity relationships. J. Funct. Foods.

[55] Costas B., Conceição L.E.C., Dias J., Novoa B., Figueras A., Afonso A. (2011). Dietary arginine and repeated handling increase disease resistance and modulate innate immune mechanisms of Senegalese sole (Solea senegalensis Kaup, 1858). Fish Shellfish Immunol..

[56] Quade M.J., Roth J.A. (1997). A rapid, direct assay to measure degranulation of bovine neutrophil primary granules. Vet. Immunol. Immunopathol..

[57] Sunyer J.O., Tort L. (1995). Natural hemolytic and bactericidal activities of sea bream Sparus aurata serum are effected by the alternative complement pathway. Vet. Immunol. Immunopathol..

[58] Costa M., Costas B., Machado M., Teixeira C., Fernández-Boo S., Sá T., Batista S., Marques A., Miranda F., Valente L.M.P. (2020). Anchovy and giant squid hydrolysates can enhance growth and the immune response of European seabass (*Dicentrarchus labrax*) fed plant-protein-based diets. Aquaculture.

[59] Sánchez-Moreno C. (2002). Review: Methods Used to Evaluate the Free Radical Scavenging Activity in Foods and Biological Systems. Food Sci. Technol. Int..

[60] Gonçalves B., Falco V., Moutinho-Pereira J., Bacelar E., Peixoto F., Correia C. (2009). Effects of Elevated CO_2_ on Grapevine (*Vitis vinifera* L.): Volatile Composition, Phenolic Content, and In Vitro Antioxidant Activity of Red Wine. J. Agric. Food Chem..

[61] Brand-Williams W., Cuvelier M.E., Berset C. (1995). Use of a free radical method to evaluate antioxidant activity. LWT Food Sci. Technol..

[62] Bradford M.M. (1976). A rapid and sensitive method for the quantitation of microgram quantities of protein utilizing the principle of protein-dye binding. Anal. Biochem..

[63] Greenwald R. (1987). CRC Handbook of Methods for Oxygen Radical Research. Free Radic. Biol. Med..

[64] Habig W.H., Pabst M.J., Jakoby W.B. (1974). Glutathione S-transferases: The first enzymatic step in mercapturic acid formation. J. Biol. Chem..

[65] Cribb A.E., Leeder J.S., Spielberg S.P. (1989). Use of a microplate reader in an assay of glutathione reductase using 5,5′-dithiobis(2-nitrobenzoic acid). Anal. Biochem..

[66] Baker M.A., Cerniglia G.J., Zaman A. (1990). Microtiter plate assay for the measurement of glutathione and glutathione disulfide in large numbers of biological samples. Anal. Biochem..

[67] Mohandas J., Marshall J.J., Duggin G.G., Horvath J.S., Tiller D.J. (1984). Differential distribution of glutathione and glutathione-related enzymes in rabbit kidney. Possible implications in analgesic nephropathy. Biochem. Pharmacol..

[68] Bird R.P., Draper H.H. (1984). [35] Comparative studies on different methods of malonaldehyde determination. Methods Enzymol.

[69] Choubert G., Blanc J.M., Vallée F. (1997). Colour measurement, using the CIELCH colour space, of muscle of rainbow trout, Oncorhynchus mykiss (Walbaum), fed astaxanthin: Effects of family, ploidy, sex, and location of reading. Aquacult. Res..

[70] Batista S., Pereira R., Oliveira B., Baião L.F., Jessen F., Tulli F., Messina M., Silva J.L., Abreu H., Valente L.M.P. (2020). Exploring the potential of seaweed *Gracilaria gracilis* and microalga *Nannochloropsis oceanica*, single or blended, as natural dietary ingredients for European seabass *Dicentrarchus labrax*. J. Appl. Phycol..

[71] MacFie H.J., Bratchell N., Greenhoff K., Vallis L.V. (1989). Designs to balance the effect of order of presentation and first-order carry-over effects in hall tests. J. Sens. Stud..

[72] Peryam D.R., Pilgrim F.J. (1957). Hedonic scale method of measuring food preferences. Food Technol..

[73] Addinsoft (2020). XLSTAT Statistical and Data Analysis Solution.

[74] Lea P., Næs T., Rødbotten M. (1997). Analysis of Variance for Sensory Data.

[75] Ares G., Barreiro C., Deliza R., Giménez A.N.A., Gàmbaro A. (2010). Application of a check-all-that-apply question to the development of chocolate milk desserts. J. Sens. Stud..

[76] Ares G., Deliza R., Barreiro C., Giménez A., Gámbaro A. (2010). Comparison of two sensory profiling techniques based on consumer perception. Food Qual. Prefer..

[77] Ares G., Varela P., Rado G., Giménez A. (2011). Are consumer profiling techniques equivalent for some product categories? The case of orange flavoured powdered drinks. Int. J. Food Sci. Technol..

[78] Anderson J.S., Sunderland R. (2002). Effect of extruder moisture and dryer processing temperature on vitamin C and E and astaxanthin stability. Aquaculture.

[79] Ortak M., Caltinoglu C., Sensoy I., Karakaya S., Mert B. (2017). Changes in functional properties and in vitro bioaccessibilities of β-carotene and lutein after extrusion processing. Int. J. Food Sci. Tech..

[80] Borsarelli C., Mercadante A.Z., Landrum J.T. (2009). Thermal and photochemical degradation of carotenoids. Carotenoids–Physical, Chemical, and Biological Functions and Properties.

[81] Sabliov C.M., Fronczek C., Astete C.E., Khachaturyan M., Khachatryan L., Leonardi C. (2009). Effects of Temperature and UV Light on Degradation of α-Tocopherol in Free and Dissolved Form. J. Am. Oil Chem. Soc..

[82] Verleyen T., Kamal-Eldin A., Dobarganes C., Verhe R., Dewettinck K., Huyghebaert A. (2001). Modeling of α-tocopherol loss and oxidation products formed during thermoxidation in triolein and tripalmitin mixtures. Lipids.

[83] Nissiotis M., Tasioula-Margari M. (2002). Changes in antioxidant concentration of virgin olive oil during thermal oxidation. Food Chem..

[84] Riaz M., Ali R. (2009). Stability of Vitamins during Extrusion. Crit. Rev. Food Sci. Nutr..

[85] Morin P., Gorman A., Lambrakis L. (2021). A literature review on vitamin retention during the extrusion of dry pet food. Anim. Feed Sci. Technol..

[86] Guerra N.B., de Almeida Melo E., Filho J.M. (2005). Antioxidant compounds from coriander (*Coriandrum sativum* L.) etheric extract. J. Food Compos. Anal..

[87] Liu D., Shi J., Colina Ibarra A., Kakuda Y., Jun Xue S. (2008). The scavenging capacity and synergistic effects of lycopene, vitamin E, vitamin C, and β-carotene mixtures on the DPPH free radical. LWT Food Sci. Technol..

[88] Cano A., Acosta M., Arnao M.B. (2000). A method to measure antioxidant activity in organic media: Application to lipophilic vitamins. Redox Rep..

[89] Prevc T., Levart A., Cigić I.K., Salobir J., Ulrih N.P., Cigić B. (2015). Rapid Estimation of Tocopherol Content in Linseed and Sunflower Oils-Reactivity and Assay. Molecules.

[90] Danet A., Waisundara V. (2021). Recent Advances in Antioxidant Capacity Assays. Antioxidants–Benefits, Sources, Mechanisms of Action.

[91] Do Q.D., Angkawijaya A.E., Tran-Nguyen P.L., Huynh L.H., Soetaredjo F.E., Ismadji S., Ju Y.-H. (2014). Effect of extraction solvent on total phenol content, total flavonoid content, and antioxidant activity of *Limnophila aromatica*. J. Food Drug Anal..

[92] van Lith R., Ameer G.A., Dziubla T., Butterfield D.A. (2016). Chapter Ten–Antioxidant Polymers as Biomaterial. Oxidative Stress and Biomaterials.

[93] Keramati S., Ferdowsi M., Zamir S.M. (2021). Compounds interactions during simultaneous biodegradation of hydrophobic n-hexane and hydrophilic methanol vapors in one- and two-liquid phase conditions. Process Saf. Environ. Prot..

[94] Carvalho C.C.C.R., Caramujo M.J. (2017). Carotenoids in Aquatic Ecosystems and Aquaculture: A Colorful Business with Implications for Human Health. Front. Mar. Sci..

[95] Kiokias S., Gordon M.H. (2004). Antioxidant Properties of Carotenoids In Vitro and In Vivo. Food Rev. Int..

[96] Sindhu E., Preethi K., Kuttan R. (2010). Antioxidant activity of carotenoid lutein in vitro and in vivo. Indian J. Exp. Biol..

[97] Mueller L., Boehm V. (2011). Antioxidant Activity of β-Carotene Compounds in Different in Vitro Assays. Molecules.

[98] Kaur A., Dhari J., Sharma O., Gupta G., Kharb V. (2012). In-vitro Anti-oxidant and Free Radical Scavenging Activity of Lycopene. Res. J. Pharm. Biol. Chem. Sci..

[99] García-Chavarría M., Lara-flores M. (2013). The use of carotenoid in aquaculture. Res. J. Fish. Hydrobiol..

[100] Williams R.J., Spencer J.P., Rice-Evans C. (2004). Flavonoids: Antioxidants or signalling molecules?. Free Radic Biol. Med..

[101] Brglez Mojzer E., Knez Hrnčič M., Škerget M., Knez Ž., Bren U. (2016). Polyphenols: Extraction Methods, Antioxidative Action, Bioavailability and Anticarcinogenic Effects. Molecules.

[102] Maqsood S., Benjakul S., Shahidi F. (2013). Emerging role of phenolic compounds as natural food additives in fish and fish products. Crit. Rev. Food Sci. Nutr..

[103] Oniszczuk T., Oniszczuk A., Gondek E., Guz L., Puk K., Kocira A., Kusz A., Kasprzak K., Wójtowicz A. (2019). Active polyphenolic compounds, nutrient contents and antioxidant capacity of extruded fish feed containing purple coneflower (*Echinacea purpurea* (L.) Moench.). Saudi J. Biol. Sci..

[104] Holdt S.L., Kraan S. (2011). Bioactive compounds in seaweed: Functional food applications and legislation. J. Appl. Phycol..

[105] BORS W., MICHEL C. (2002). Chemistry of the Antioxidant Effect of Polyphenols. Ann. N. Y. Acad. Sci..

[106] Lu S.C. (2013). Glutathione synthesis. Biochim. Biophys. Acta Gen. Subj..

[107] Kurutas E.B. (2015). The importance of antioxidants which play the role in cellular response against oxidative/nitrosative stress: Current state. Nutr. J..

[108] Bragadóttir M., Pálmadóttir H., Kristbergsson K. (2001). Endogenous Antioxidants in Fish. Master’s Thesis.

[109] Lizárraga-Velázquez C.E., Hernández C., González-Aguilar G.A., Heredia J.B. (2019). Effect of dietary intake of phenolic compounds from mango peel extract on growth, lipid peroxidation and antioxidant enzyme activities in zebrafish (*Danio rerio*). Lat. Am. J. Aquat. Res..

[110] Castenmiller J.J.M., West C.E. (1998). Bioavailability and Bioconversion of Carotenoids. Annu. Rev. Nutr..

[111] Mourente G., Díaz-Salvago E., Bell J.G., Tocher D.R. (2002). Increased activities of hepatic antioxidant defence enzymes in juvenile gilthead sea bream (*Sparus aurata* L.) fed dietary oxidised oil: Attenuation by dietary vitamin E. Aquaculture.

[112] Gao J., Koshio S., Ishikawa M., Yokoyama S., Mamauag R.E.P., Han Y. (2012). Effects of dietary oxidized fish oil with vitamin E supplementation on growth performance and reduction of lipid peroxidation in tissues and blood of red sea bream Pagrus major. Aquaculture.

[113] Palace V.P., Majewski H.S., Klaverkamp J.F. (1993). Interactions among Antioxidant Defenses in Liver of Rainbow Trout (Oncorhynchus mykiss) Exposed to Cadmium. Can. J. Fish. Aquat. Sci..

[114] Kelestemur G.T., Seven P.T., Yilmaz S. (2012). Effects of dietary propolis and vitamin E on growth performance and antioxidant status in blood of juvenile Rainbow trout, Oncorhynchus mykiss (Teleostei: Salmoniformes) under different flow rates. Zoologia.

[115] Lygren B., Hjeltnes B., Waagbø R. (2002). Immune response and disease resistance in Atlantic salmon (*Salmo salar* L.) fed three levels of dietary vitamin E and the effect of vaccination on the liver status of antioxidant vitamins. Aquacult. Int..

[116] Silva R.F., Sanchéz Vasquéz F.J., Martinéz López F.J., Sánchez Vázquez F.J., Muñoz-Cueto J.A. (2014). Nutrition and Dietary Selection. Biology of European Sea Bass.

[117] Abdel-Tawwab M., Samir F., Abd El-Naby A.S., Monier M.N. (2018). Antioxidative and immunostimulatory effect of dietary cinnamon nanoparticles on the performance of Nile tilapia, *Oreochromis niloticus* (L.) and its susceptibility to hypoxia stress and Aeromonas hydrophila infection. Fish Shellfish Immunol..

[118] Nakajima K., Yoshie-Stark Y., Ogushi M. (2009). Comparison of ACE inhibitory and DPPH radical scavenging activities of fish muscle hydrolysates. Food Chem..

[119] Sikorski Z.E., Olley J., Kostuch S., Olcott H.S. (1976). Protein changes in frozen fish. Crit. Rev. Food Sci. Nutr..

[120] Turrens J.F. (2003). Mitochondrial formation of reactive oxygen species. J. Physiol..

[121] Araújo M., Rema P., Sousa-Pinto I., Cunha L.M., Peixoto M.J., Pires M.A., Seixas F., Brotas V., Beltrán C., Valente L.M.P. (2016). Dietary inclusion of IMTA-cultivated Gracilaria vermiculophylla in rainbow trout (*Oncorhynchus mykiss*) diets: Effects on growth, intestinal morphology, tissue pigmentation, and immunological response. J. Appl. Phycol..

[122] Tibaldi E., Chini Zittelli G., Parisi G., Bruno M., Giorgi G., Tulli F., Venturini S., Tredici M.R., Poli B.M. (2015). Growth performance and quality traits of European sea bass (*D. labrax*) fed diets including increasing levels of freeze-dried Isochrysis sp. (T-ISO) biomass as a source of protein and n-3 long chain PUFA in partial substitution of fish derivatives. Aquaculture.

[123] Spence C., Levitan C.A., Shankar M.U., Zampini M. (2010). Does Food Color Influence Taste and Flavor Perception in Humans?. Chemosens. Percept..

[124] Roth B., Birkeland S., Oyarzun F. (2009). Stunning, pre slaughter and filleting conditions of Atlantic salmon and subsequent effect on flesh quality on fresh and smoked fillets. Aquaculture.

